# Potent Xanthine Oxidase Inhibitory Activity of Constituents of *Agastache rugosa* (Fisch. and C.A.Mey.) Kuntze

**DOI:** 10.3390/foods12030573

**Published:** 2023-01-28

**Authors:** Heung Joo Yuk, Hyung Won Ryu, Dong-Seon Kim

**Affiliations:** 1 KM Science Research Division, Korea Institute of Oriental Medicine (KIOM), Daejeon 34054, Republic of Korea; 2 Natural Medicine Research Center, Korea Research Institute of Bioscience and Biotechnology (KRIBB), Cheong-ju 28116, Republic of Korea

**Keywords:** *Agastache rugosa*, xanthine oxidase, acacetin, gout, hyperuricemia

## Abstract

The aerial parts of *Agastache rugosa* are used as a food material and traditional medicine in Asia. A 50% ethanol extract exhibited potent xanthine oxidase (XO) inhibitory activity (IC_50_ = 32.4 µg/mL). To investigate the major components responsible for this effect, seven known compounds were identified from *A. rugosa*; among these, salvianolic acid B (**2**) was isolated from this plant for the first time. Moreover, acacetin (**7**) exhibited the most potent inhibitory activity with an IC_50_ value of 0.58 µM, lower than that of allopurinol (IC_50_ = 4.2 µM), which is commonly used as a XO inhibitor. Comparative activity screening revealed that the C6-bonded monosaccharides (**3**) or sugars substituted with acetyl or malonyl groups (**4**–**6**) are critical for XO inhibition when converted to aglycone (**7**). The most potent inhibitor (**7**) in the *A. rugosa* extract (ARE) exhibited mixed-type inhibition kinetics and reversible inhibition toward XO. Furthermore, the hydrolysis of ARE almost converted to an inhibitor (**7**), which displayed the highest efficacy; UPLC-qTof MS revealed an increased content, up to five times more compared with that before treatment. This study will contribute to the enhancement in the industrial value of ARE hydrolysates as a functional ingredient and natural drug toward the management of hyperuricemia and treatment of gout.

## 1. Introduction

The main causes of hyperuricemia are the over-production of uric acid, insufficient excretion in the urine, or a combination of both [1,2]. When blood levels of uric acid are high, uric acid enters the joints and crystallizes, causing joint inflammation; the inflammation causes swelling and pain in the joints, leading to gout and gouty arthritis which are excruciatingly painful [3,4]. Xanthine oxidase (XO, EC 1.17.3.2) is an important enzyme in the purine catabolic pathway in the body; it is present in significant concentrations in the gastrointestinal tract. XO oxidizes hypoxanthine into xanthine followed by xanthine conversion to uric acid [5]. Thus, XO inhibitors that block uric acid synthesis can play an important role in preventing hyperuricemia. The administration of allopurinol, a well-known representative XO inhibitor, is one of the clinical treatment methods for hyperuricemia and chronic gout [6]. However, despite the excellent efficacy of allopurinol, side effects such as nausea, diarrhea, or drowsiness may occur in general. In particular, in cases of renal dysfunction, it can be fatal; hypersensitivity syndromes such as gastrointestinal disorder, skin rash, or fever may also occur when diuretics are administered [7]. Therefore, the development of potential XO inhibitors from natural materials that increase effectiveness, reduce side effects, and guarantee safety is highly desirable. One such natural resource is *Agastache rugosa* (Fisch. and C.A.Mey.) Kuntze.

*A. rugosa* is a perennial plant belonging to the Lamiales order Lamiaceae family; it is known to form a colony and has high growth power even in barren mountain fields [8]. Geographically, it is mainly distributed in Northeast Asia, China, Japan, and Korea, where the leaves are used as spices and the flowers as tea. Moreover, in traditional medicine, the aerial part is used as an important source of medicinal resources [9,10]. It has been reported that the main use of *A. rugosa* is for treating vomiting, fever, diarrhea, anxiety, halitosis, nausea, and headaches [11,12]. Previous studies have revealed that the *A. rugosa* extract (ARE) displays an anti-osteoporotic effect on estrogen-deficiency-induced osteoporosis in animal models and its mechanism of inhibition on osteoclast differentiation has been elucidated [13]. There is also pharmacological evidence that ARE exhibits anti-inflammatory, anti-tumor, and anti-atherogenic effects [14,15]. These effects may be due to the proliferation of active compounds in ARE.

ARE has been reported to contain various types of functional metabolites including phenolic acids, triterpenes, essential oils, and flavonoids [16,17]. Many studies have reported that components isolated from *A. rugosa* contribute to bioactive effects; however, no detailed research regarding potential XO inhibitors has been performed. In particular, among the flavonoids, apigenin is known to be a strong XO inhibitor, and structurally very similar components of *A. rugosa* have been reported in the literature [18]. Thus, this study intends to confirm the XO inhibitory effect.

Therefore, to support the use of safe and highly potent natural inhibitors for the prevention, symptom relief, and treatment of hyperuricemia, we isolated the key constituents responsible for the XO inhibitory effect and investigated the mechanism of inhibition by kinetic mode analysis. In addition, for industrial use purposes, ARE was converted to a material that maximized the active ingredients through hydrolysis.

## 2. Materials and Methods

### 2.1. Plant Materials and Sample Preparation

The aerial part of *A. rugosa* is called Kwakhayang and was purchased from the oriental herbal market (Omniherb, Daegu, Republic of Korea) (http://www.omniherb.com/, accessed on 1 September 2022) that only handles herbs certified by the Korean Pharmacopoeia. A sample was ground to a powder state to ensure uniform extraction and stored at −80 °C in a cryogenic freezer until further experiment. A total of 5 different solvents for extraction were used, ethanol (EtOH), 70% EtOH in water (EtOH/H_2_O (7/3, *v*/*v*)), 50% EtOH, 30% EtOH, and distilled water (H_2_O). To prepare the various extracts for the activity-guided fractionation, 2 g of crushed aerial parts were immersed in 40 mL of each solvent and were extracted ultrasonically for 1 h at 25 °C. For hydrolysis, 2 mg of 50% ARE was treated with 1 mL of 50 mM phosphate buffer (pH 6.8) with 0.2 units of β-glucosidase. The reaction mixture was incubated at 37 °C for 2 h to completely hydrolyze the glucose moiety with the β form [19]. The samples were centrifuged at 4000× *g* for 5 min, filtered through a 0.22 μm PTFE membrane filter, and used for liquid chromatography analyses and enzymatic inhibition evaluations. For quantitative analyses, calibration curves were plotted with high linearity (*r*^2^ > 0.998) for 6 different concentrations (100 μg, 50 μg, 10 μg, 5 μg, 1 μg, and 0.1 μg/mL). HPLC-grade solvents such as water, acetonitrile (ACN), and ethanol were purchased from J. T. Baker (Phillipsburg, Pennsylvania, USA). β-Glucosidase from almonds, XO from bovine milk, xanthine, allopurinol, apigenin, sodium pyrophosphate, dimethyl sulfoxide (DMSO), and formic acid (FA) of MS-grade were purchased from Sigma-Aldrich (St. Louis, MO, USA).

### 2.2. Instruments

The ^1^H and ^13^C nuclear magnetic resonance (NMR) spectra were obtained on a Bruker AM 400, using DMSO-*d*_6_ and CD_3_OD with TMS (Andover, MA, USA) as the internal standard. Electrospray ionization (ESI) high-resolution (HR) mass spectrometry (MS) spectra were analyzed on a quadrupole time-of-flight mass spectrometer (qTof Premier^TM^, Waters Corp., Milford, MA, USA). The ultra-performance liquid chromatography (UPLC) system equipped with a UV detector, binary gradient module, and an auto sampler was from Waters Corp. (Milford, MA, USA). Separation and purification of single compounds were performed using a medium-pressure liquid chromatography (MPLC) and recycling HPLC (YMC Co., Ltd., Kyoto, Japan) with a reversed-phase (RP) cartridge and preparative RP column (250 mm × 20 mm, i.d. 5 μm). All the enzymatic assay experiments were performed on a multi-mode microplate reader, SpectraMax M2 series (Molecular device, San Jose, CA, USA).

### 2.3. Extraction, Fractionation, and Isolation of A. rugosa

The air-dried aerial part (600 g) of *A. rugosa* was ground using a blender (SHMFP-40000; HANIL Co., Ltd., Seoul, South Korea) and extracted twice with 50% EtOH (12 L) for 4 days each at room temperature (25 °C). The filtered and combined extract was evaporated, resulting in 67.8 g (yield 11.3%) of crude extract. A portion of the sample (50 g) of this extract was subjected to column chromatography (CC) on a Diaion HP20SS (12 × 50 cm, 1.2 kg) eluted with MeOH/H_2_O mixed solvents (10:90 (2 L), 30:70 (2 L), 50:50 (4 L), 70:30 (4 L), 90:10 (2 L), and 100:0 (2 L)), to provide seven fractions (A–G). Fraction B (3.2 g) was fractionated on an C18 column cartridge using MPLC with a linear gradient of 35–50% MeOH/H_2_O at an 18 mL/min flow rate to afford 5 fractions (B1–B4). The B3 subfraction, enriched with **1** and **2,** was purified by preparative HPLC using 50% MeOH as mobile phase and afforded **1** (124 mg) and **2** (2.7 mg). Fractions C–E (8.9 g) enriched with **3**–**6** were fractionated via RP-MPLC with a linear gradient of 45–60% MeOH/H_2_O to give fractions C1–C9. The C3 subfraction, enriched with **3** and **4**, was further purified by recycling preparative HPLC using 55% MeOH in H_2_O and afforded **3** (184 mg) and **4** (92 mg). Subsequent separation of subfractions (C5–C7), enriched with **5** and **6**, were combined and further purified by recycling preparative HPLC mode using 40% ACN in H_2_O as the mobile phase to afford **5** (5.2 mg) and **6** (7.4 mg). Fraction F (0.7 g) enriched with **7** on Sephadex LH-20 with 60% ACN as the eluent yielded **7** (16.4 mg). All isolated compounds (**1**–**7**) were identified based on the following physicochemical and spectroscopic data [16,20].

*Rosmarinic acid* (**1**). White powder; *λ*_max_ (MeOH) 200, 329 nm; HR-ESI-MS, *m*/*z* 359.0777 [M–H]^−^ (calcd. for C_18_H_15_O_8_ 359.0767). ^1^H NMR and ^13^C NMR data, see Supplementary Materials.

*Salvianolic acid B* (**2**). Amorphous white powder; [α]^20^_D_ +94.0 (c 0.7, CH_3_OH); *λ*_max_ (MeOH) 254, 287, 329 nm; HR-ESI-MS, *m*/*z* 717.1453 [M–H]^−^ (calcd. for C_36_H_30_O_16_ 717.1456). ^1^H NMR (500 MHz, CD_3_OD) δ_H_ 3.10-2.95 (4H, m, H-7″α, 7″β, 7‴α, 7‴β), 4.40 (1H, d, *J* = 4.4 Hz, H-3), 5.11–5.20 (2H, m, H-8″, 8‴), 5.83 (1H, d, *J* = 4.4 Hz, H-2), 6.29 (1H, d, *J* = 16.0 Hz, H-11‴), 6.46 (1H, dd, *J* = 2.0, 8.0 Hz, H-6″), 6.57-6.65 (8H, m, H-6‴, 6′, 5′, 5‴, 2″, 2′, 2‴, 5″), 6.76 (1H, d, *J* = 8.4 Hz, H-6), 7.17 (1H, d, *J* = 16.0 Hz, H-5), 7.68 (1H, d, *J* = 16.0 Hz, H-12‴). ^13^C NMR (125 MHz, CD_3_OD) δC 172.4 (C-9″), 170.1 (C-10″), 168.1 (C-9‴), 166.9 (C-10‴), 148.9 (C-8), 146.5 (C-7), 146.4 (C-3‴, 4‴), 145.9 (C-3′, 4′), 145.1 (C-3″,4″), 133.7 (C-1′), 129.2 (C-1″), 128.7 (C-1‴), 126.2 (C-9), 124.7 (C-4), 144.1 (C-12‴), 121.9 (C-5, 6″, 6‴), 118.3 (C-6, 6′), 117.5 (C-2″, 2‴), 116.3 (C-5′, 5″, 5‴, 11‴), 113.2 (C-2′), 88.8 (C-2), 74.4 (C-8″), 74.4 (C-8″), 57.2 (C-3), 37.8 (C-7‴), and 37.2 (C-7″).

*Tilianin* (**3**). White-yellowish powder; *λ*_max_ (MeOH) 267, 332 nm; HR-ESI-MS, *m*/*z* 447.1286 [M+H]^+^ (calcd. for C_22_H_23_O_10_ 447.1291). ^1^H NMR and ^13^C NMR data, see Supplementary Materials.

*Acacetin 7-O-(6-O-malonyl)-β-D-glucoside* (**4**). Yellowish amorphous powder; *λ*_max_ (MeOH) 267, 332 nm; HR-ESI-MS, *m*/*z* 533.1293 [M+H]^+^ (calcd. for C_25_H_25_O_13_ 533.1295). ^1^H NMR and ^13^C NMR data, see Supplementary Materials.

*Acacetin 7-O-(2″-O-acetyl)-β-D-glucoside* (**5**). Yellowish crystalline powder; *λ*_max_ (MeOH) 267, 332 nm; HR-ESI-MS, *m*/*z* 489.1394 [M+H]^+^ (calcd. for C_24_H_25_O_11_ 489.1397). ^1^H NMR and ^13^C NMR data, see Supplementary Materials.

*Acacetin 7-O-(2″-O-acetyl-6″-O-malonyl)-β-D-glucoside* (**6**). Pale-brown amorphous powder; *λ*_max_ (MeOH) 267, 332 nm; HR-ESI-MS, *m*/*z* 575.1399 [M+H]^+^ (calcd. for C_27_H_27_O_14_ 575.1401). ^1^H NMR and ^13^C NMR data, see Supplementary Materials.

*Acacetin* (**7**). White-yellowish powder; *λ*_max_ (MeOH) 267, 332 nm; HR-ESI-MS, *m*/*z* 285.0755 [M+H]^+^ (calcd. for C_16_H_13_O_5_ 285.0763). ^1^H NMR and ^13^C NMR data, see Supplementary Materials.

### 2.4. UPLC-qTof Mass Spectrometry Analysis

The identification of the main component peaks was performed by a UPLC-coupled PDA detector. An amount of 2.0 μL of each sample was injected into a BEH C18 column (i.d., 1.7 μm, 100 × 2.1 mm) at a flow rate of 0.4 mL (per min) and eluted using a chromatographic gradient of 2 mobile phases (A: H_2_O containing 0.1% FA; B: ACN containing 0.1% FA). The linear gradient elution was optimized as follows: 0 min, 10% B; 0–1 min, 10% B; 1–8 min, 10–40% B; 8–11 min, 40–90% B; 11–11.3 min, 90–100% B; 11.3–13.3 min, 100% B; 13.3–13.4 min, 100–10% B; and 13.4–15 min, return to initial elution condition of 10% B. The qTof MS was operated in two different ion modes (negative and positive) under the following conditions: cone voltage 30 V, capillary voltage 2.5 kV, source temperature 110 °C, and desolvation temperature 400 °C. A sprayer with a calibration (lock MS) standard solution of leucine-enkephalin ([M–H]^−^ *m*/*z* 554.2615, [M+H]^+^ *m*/*z* 556.2771) was used. The UV chromatograms were obtained using Empower 2, and full scan MS data and MS^2^ spectra were collected using the MassLynx software.

### 2.5. XO Inhibitory Activity and XO Kinetic Assay

The bovine milk XO activity was assayed following the previously published procedures [21,22]. Allopurinol and apigenin were used as the positive controls for the enzyme assay protocol. The compounds (**1**–**7**) for evaluation of inhibitory effects were dissolved in DMSO at 4 mM and further diluted at various concentrations. First, 125 µL of 100 mM sodium pyrophosphate buffer (HCl, pH 7.5), 30 µL of 0.1 units XO enzyme, 5 µL of test samples, and 40 µL of the substrate (0.5 mM xanthine) were mixed. The reaction mixture (200 µL) in a 300 µL well plate was incubated at 37 °C and the UV absorbance at 295 nm was screened every 1 min for 15 min. The inhibition mechanism and constants (*K*_m_, *V*_max_, and *K*_i_) of a binding site for the inhibitor to a free enzyme (*K*_I_) or an enzyme–substrate complex (*K*_IS_) were determined using the Lineweaver–Burk and Dixon plots [23].

1/*V* = *K*_m_/*V*_max_ (1 + [I]/*K*_I_) 1/S + 1/*V*_max_ (1)

Slope = *K*_m_/*K*_I_
*V*_max_ [I] + *K*_m_/*V*_max_ (2)

Intercept = 1/*K*_IS_
*V*_max_ [I] + 1/*V*_max_ (3)

### 2.6. Statistical Analysis

All measurements of the XO enzyme activities and individual compound contents were repeated in triplicate and the data were expressed as mean ± SD (standard deviations) using SigmaPlot version 10.0 (Systat Software Inc., San Jose, CA, USA).

## 3. Results and Discussion

### 3.1. Bioassay-Guided Isolation and Identification of Compounds

The ARE was prepared using five different polar solvents (EtOH, 70% EtOH, 50% EtOH, 30% EtOH, and H_2_O) and tested for their XO inhibitory activity. The degree of in vitro enzyme inhibition was measured spectrophotometrically by determining the uric acid formation in absorbance at 295 nm, using xanthine (C_5_H_4_N_4_O_2_) as the substrate. All extracts investigated, except the H_2_O extract, showed more than 50% inhibitory activity against XO at 125 μg/mL (Table 1).

In particular, the 50% EtOH solvent (IC_50_ = 32.4 μg/mL) extracted the maximum XO inhibitory substances. In fact, the 50% ARE exhibited inhibitory effects statistically similar to those of the 70% ARE (IC_50_ = 31.9 μg/mL). The 50% EtOH extract was selected considering its industrial use parameters, such as the unit cost of EtOH, ease of powdering, and especially the point with the highest yield (11.3%). The high potency of the 50% EtOH extract via activity-guided fractionation encouraged us to identify compounds responsible for its XO inhibitory effects.

**Figure 1:**
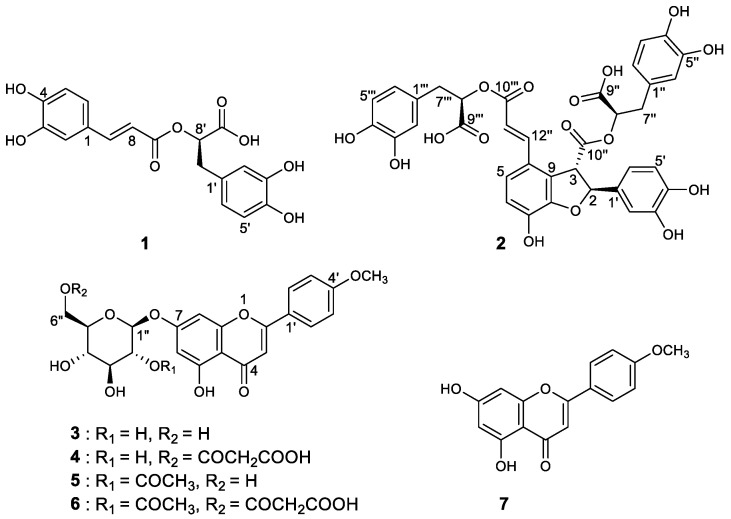
Structures of compounds **1**–**7** isolated from *A. rugosa*.

As a result of separation and purification through repeated CC, seven XO inhibitory phenolic compounds (**1** and **2**) and flavonoids (**3**–**7**) were isolated from 50% ARE (Figure 1). The isolated compounds **1**–**7** were identified as rosmarinic acid (**1**), salvianolic acid B (**2**), tilianin (**3**), acacetin 7-*O*-(6″-*O*-malonyl)-β-D-glucopyranoside (**4**), acacetin 7-*O*-(2″-*O*-acetyl)-β-D-glucopyranoside (**5**), acacetin 7-*O*-(2″-*O*-acetyl-6″-*O*-malonyl)-β-D-glucopyranoside (**6**), and acacetin (**7**) by comparing our spectroscopic analysis data (Supplementary Materials, Figures S1–S12 and Table S1) with previously reported data [16,20]. Interestingly, the trace compound salvianolic acid B (**2**) was first identified in the ARE. The structure of **2** was elucidated using established spectroscopic analysis data of NMR and ESI-HR-MS. Among the isolated compounds, **7** exhibited the highest XO inhibitory effect with a molecular formula of C_16_H_12_O_5_ as established by the [M+H]^+^ ion at *m*/*z* 285.0755 (calcd. 285.0763) in the ESI-HR-MS analysis using qTof MS. The ^13^C-NMR data enabled carbons corresponding to seven C–C double bonds and one carbonyl group to be identified, thus accounting for 8 of the 11 degrees of unsaturation. The extra three degrees of unsaturation were ascribed to three aromatic rings. In the ^1^H-NMR spectrum, the presence of seven aromatic protons indicated a trisubstituted typical flavonoid skeleton. Four proton values for ring B protons appeared in the form of two sets of doublets (*J* = 8.8 Hz) at δ 8.02 (2H, H-2′, H-6′) and 7.10 (2H, H-3′, H-5′) with their corresponding carbons at 128.3 and 114.6 ppm, respectively. Thus, **7** was identified as acacetin.

*A. rugosa* is a well-known aromatic plant; most studies have focused on its volatile components including essential oils such as estragole, limonene, menthone, and pulegone. Until recently, Hong et al. reported that the essential oil components of *A. rugosa* help improving human concentration and freshness of the human brain [24]. Furthermore, the non-volatile polar components of *A. rugosa* are important, including acacetin (**7**) and their derivatives because they induce a very strong effect on the XO inhibitory activity according to our study.

### 3.2. XO Inhibitory Activity of Identified Compounds

**Figure 2:**
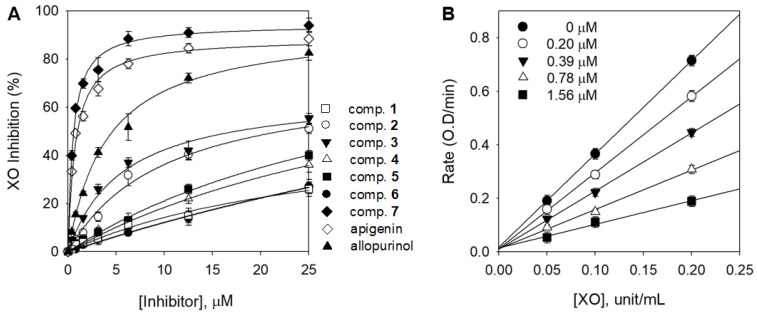
(**A**) Inhibitory effects of compounds (**1**–**7**) on the activity of XO based on the oxidation of xanthine to uric acid. (**B**) Reversible relationship of the catalytic activity of XO between enzyme concentrations in compound **7** at different concentrations.

All isolated compounds **1**–**7** were evaluated for their inhibitory effects on XO activity and their inhibitory mechanism was derived through kinetic analysis. Phenolic acid derivatives (**1** and **2**), flavone glycosides (**3**, **4**, **5**, and **6**), and flavone aglycone (**7**) had a concentration-dependent effect (Figure 2A). Nile et al. reported that phenolic acid derivatives containing caffeic acid, ferulic acid, and sinapic acid are effective against XO [25]. Lin et al. also reported flavonoids such as apigenin, luteoline, kaempferol, and quercetin had potent XO inhibitory effects [18]. However, the XO inhibitory effect showed that phenolic acids and flavonoid compounds were inactive (IC_50_: >25 μM) except for acacetin (**7**), a flavonoid aglycone (Table 1).

Compound **7** exhibited considerably higher inhibitory activity (75.5%) at the same concentration of 5 μM compared with apigenin (67.2%) and allopurinol (51.9%), which were used as positive controls. Acacetin (**7**) (IC_50_ = 0.58 µM), which is more effective than apigenin (IC_50_ = 0.87 µM) and is known to have the highest inhibitory activity among flavonoids, was revealed for the first time. It appears that inhibition was higher when (**7**) has a methoxy group instead of a hydroxyl group at the 4′-position. These results are in agreement with those reported by Liu, in which some flavonoids have higher inhibitory activity when they are substituted with methoxy groups [26]. Acacetin glycosides (**3**–**6**) showed significantly lower inhibitory activities, between 26.4 and >100 µM, compared with aglycone **7**. This difference may be due to the presence of the monosaccharide glucoside and the additional malonyl, acetyl, or acetyl–malonyl on the sugar moiety. Nguyen et al. used luteolin and luteolin 7-*O*-glucoside and revealed that a glycoside is more than 10 times weaker than its aglycone [27]. Biochemically, the XO inhibition is associated with the hydrogen bonding between the phenolic hydroxyl functionalities of the substrates and the abundant amino groups in the peptide chain of the enzyme. Therefore, the glycosyl substitution of the A-ring C6 of acacetin (**3**–**6**) more effectively prevents the binding to the XO active site than aglycone **7** [28]. As shown in Figure 2B, to confirm the reversible inhibition caused by (**7**), a plot of the various inhibitor concentrations (0 μM, 0.20 μM, 0.39 μM, 0.78 μM, and 1.56 μM) versus its activity at different enzyme concentrations (0 unit/mL, 0.05 unit/mL, 0.1 unit/mL, and 0.2 unit/mL) was analyzed.

**Figure 3:**
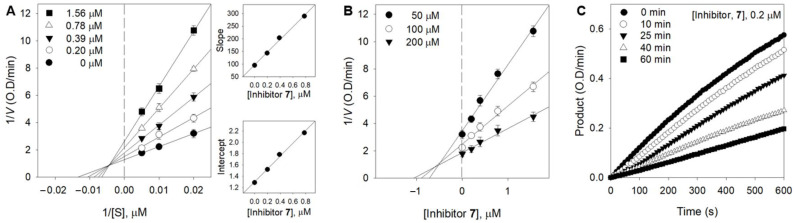
Enzymatic kinetics of XO inhibition by **7**. (**A**) Lineweaver–Burk plots of **7**. (Inset) The secondary plots of the slope and the intercept of the straight lines versus the concentration of **3** throughout Equations (1)–(3) are shown. (**B**) Dixon plots of **7**. The graphical symbols are substrate concentrations (50 µM, ●; 100 µM, ○; 200 µM, ▼). (**C**) Inhibition as a function of preincubation time for **7** at 0.2 µM.

The kinetic behaviors of the oxidation of xanthine catalyzed by XO at different concentrations of inhibitor were analyzed using both Lineweaver–Burk (Figure 3A) and Dixon plots (Figure 3B). Figure 3A shows the mixed-type inhibitor, indicating that *V*_max_ was decreased, whereas *K*_m_ increased with the increasing concentration of **7** from 0 to 1.56 μM. The two possible inhibition mechanisms were investigated to reveal which one gives this kinetic profile. The mechanisms of mixed inhibition can be investigated through the changes of the substrate with inhibitor followed by measurements of the residual enzyme–substrate complex. Parameters of free enzyme (*K*_I_) and the enzyme–substrate complex (*K*_IS_) can be fitted to Equations (2) and (3) [29]. The secondary plots of the straight lines of the slope and intercept showed that *K*_I_ (0.57 μM) was much less than *K*_IS_ (1.14 μM). It is thus reasonable to conclude that **7** is allowed as mixed type I when the inhibitor prefers the free enzyme over the substrate–enzyme complex. The values of *K*_IS_ were approximately two times greater, suggesting a weaker binding of **7** to the XO-xanthine intermediate. This indicates that the inhibition mechanism is competitive and predominated over noncompetitive. The *K*_i_ value of **7** was determined to be 0.61 μM by the Dixon plots (Figure 3B). Further analysis of the progress of the inhibition was undertaken by preincubating the enzyme with **7**, prior to the addition of the substrate. The enzyme lost no more than 10% of its activity across the whole assay due to intrinsic decay (control). When the enzyme was preincubated with an inhibitor (0.2 μM) for a period of time, a sustained decrease in activity was observed, demonstrating that it has a time-dependent inhibitory behavior (Figure 3C).

### 3.3. UPLC-qTof Mass Spectrometry Profiles

**Figure 4:**
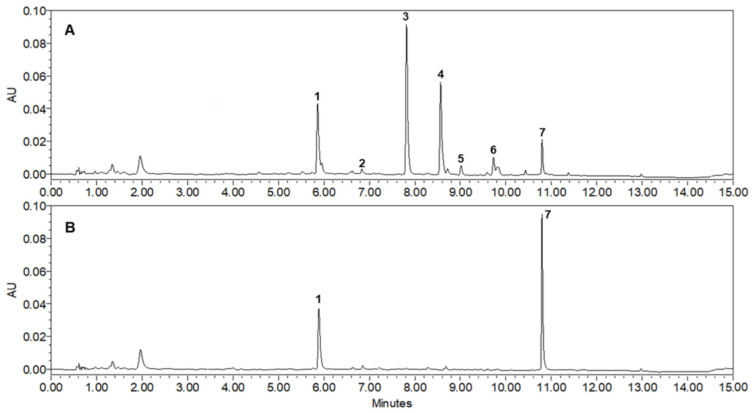
UPLC-PDA chromatograms of 50% EtOH extract from the aerial parts of *A. rugosa* (265 nm): (**A**) Control of 50% ARE; (**B**) hydrolysis of 50% ARE with 0.2 units of β-glucosidase. The order of peaks (**1**–**7**) is: **1**. rosmarinic acid, **2**. salvianolic acid B, **3**. tilianin, **4**. acacetin 7-*O*-(6″-*O*-malonyl)-β-D-glucopyranoside, **5**. acacetin 7-*O*-(2″-*O*-acetyl)-β-D-glucopyranoside, **6**. acacetin 7-*O*-(2″-*O*-acetyl-6″-*O*-malonyl)-β-D-glucopyranoside, and **7**. acacetin.

To increase the industrial applicability of the ARE, the extraction conditions were optimized and the content of potent XO inhibitor (**7**), which had the highest inhibitory activity, was analyzed using UPLC-qTof MS. The extracts for quantitative analysis were repeatedly sonicated for 30 min at 25 °C using 5 different solvents (EtOH, 70% EtOH, 50% EtOH, 30% EtOH, and H_2_O) and the most abundant peaks (**1**–**7**) were identified in 50% ARE. As shown in Table 1, the 50% ARE showing the highest inhibitory activity on XO showed the highest yield of 11.3% compared with the other extracts; the content of each peak was also the most abundant. The contents of 7 isolated compounds in the different ARE were analyzed using a UPLC at 265 nm, the maximum UV wavelength of flavonoid derivatives. All detected peaks showed molecular ions with masses consistent with those of our isolated compounds (**1**, *m*/*z* 359.0777 [M–H]^−^; **2**, *m*/*z* 717.1453 [M–H]^−^; **3**, *m*/*z* 447.1286 [M+H]^+^; **4**, *m*/*z* 533.1293 [M+H]^+^; **5**, *m*/*z* 489.1394 [M+H]^+^; **6**, *m*/*z* 575.1399 [M+H]^+^; **7**, *m*/*z* 285.0755 [M+H]^+^). The standard curve demonstrated a highly validated correlation coefficient (*r*^2^ > 0.998) and was linear and reproducible. As a result of the quantitative analysis, it was confirmed that the contents of **1**–**7** in the extracted 50% EtOH were 4.05 mg/g, 0.06 mg/g, 9.04 mg/g, 5.12 mg/g, 0.37 mg/g, 0.45 mg/g, and 1.96 mg/g per dry sample, respectively. Table 2 shows the retention time (*t*_R_), UV absorption maxima (*λ*_max_), and MS spectral data, including molecular ion and elemental composition of the peaks (**1**–**7**) [16,20]. A PDA chromatogram of 50% ARE (Figure 4A) shows that acacetin (**7**), which has the highest inhibitory activity on XO, is accumulated as a monosaccharide glycoside (**3**) or mostly substituted with units of malonyl-glc (**4**), acetyl-glc (**5**), or acetyl-malonyl-glc (**6**). Thus, to hydrolyze glycoside compounds (**3**–**6**) to aglycone **7** (Figure 4B), 50% ARE was treated with β-glucosidase at 37 °C for 2 h. Gratifyingly, the hydrolyzed 50% ARE exhibited 5-fold higher XO inhibition than the control ARE with IC_50_ = 6.2 µg/mL and IC_50_ = 32.4 µg/mL, respectively. Furthermore, it was almost converted to inhibitor **7** (10.2 mg/g per dry sample) and the content increased by more than 5 times compared with that before treatment (1.96 mg/g per dry sample). According to Kim et al., the acacetin (**7**) content increased to 3 mg/g per dry sample level when ARE was treated with lactic acid bacteria [30]. However, such an increase in the **7** content caused by bacterial treatment indicates that the selective hydrolysis of only the glucose moiety with the β form or its complete conversion to an aglycone is difficult. In addition, regarding the industrial use of the hydrolysis extract of 50% ARE, more research is needed involving acid hydrolysis in addition to treatment with enzymes or bacteria because acid hydrolysis is more competitive in terms of manufacturing cost.

## 4. Conclusions

The ARE exhibited a significant inhibitory effect on XO in vitro. Importantly, it was revealed for the first time that the most potent inhibitor was acacetin (**7**) with a low micromolar inhibition (IC_50_ = 0.58 μM) against XO. The evaluation of the detailed kinetic analysis using double-reciprocal plots revealed that **7** exhibited a mixed-type inhibition (*K*_i_ = 0.61 μM). The index/active ingredient information was provided, and rapid analysis methods for standardization and quality control of the ARE were conducted. In addition, this work presents the possibility of developing a material that maximizes the active ingredient through hydrolysis; we expect that the ARE can be industrially used as a natural drug for the management of hyperuricemia and treatment of gout.

## Figures and Tables

**Table 1:** XO Inhibitory effects of different solvent extracts and isolated compounds **1**–**7** in aerial parts of *A. rugosa*.

Compound	Extraction Yield (%) ^1^	Xanthine Oxidase		
		IC_50_	Inhibition % ^2^	Kinetic Mode (*K*_i_, µM)
EtOH	3.9	54.2 ± 0.9 µg/mL	71.8 ± 1.3	-
70% EtOH	10.1	31.9 ± 1.4 µg/mL	88.4 ± 1.1	-
50% EtOH	11.3	32.4 ± 1.2 µg/mL	86.2 ± 1.2	-
30% EtOH	9.5	64.8 ± 1.1 µg/mL	63.7 ± 1.1	-
H_2_O	12.0	>250 µg/mL	31.8 ± 1.6	-
1	-	>100 µM	27.2 ± 1.5	NT ^3^
2	-	30.7 ± 0.8 µM	46.1 ± 1.2	NT
3	-	26.4 ± 0.6 µM	49.3 ± 1.5	NT
4	-	80.6 ± 0.7 µM	36.2 ± 1.4	NT
5	-	74.5 ± 0.9 µM	40.7 ± 1.5	NT
6	-	>100 µM	26.2 ± 0.9	NT
7	-	0.58 ± 0.5 µM	94.5 ± 1.4	Mixed (0.61)
Apigenin	-	0.87 ± µM	88.6 ± 1.2	Competitive
Allopurinol	-	4.2 ± µM	82.5 ± 1.2	Competitive

^1^ Extraction yields are given as mg/g dry weight. ^2^ Sample concentration: 125 µg/mL (ppm) for the extract and 25 µM for the compound. ^3^ NT is not tested.

**Table 2:** Spectroscopic characteristics and contents (mg/g) of the seven investigated compounds from the aerial parts of *A. rugosa*.

Peak	*t*_R_	*λ*_max_	Dried Aerial Parts(mg/g) ^a^		Molecular Ion [M–H]^−^/[M+H]^+^	Elemental Composition	Identity
(no.)	(min)	(nm)	50% ARE	Hydrolysis of 50%ARE	(*m*/*z*)	(ppm Error)	
1	5.94	200,329	4.05	3.82	359.0777 [M–H]^−^	C_18_H_16_O_8_ (2.8)	Rosmarinic acid
2	6.85	287,329	0.06	0.05	717.1453 [M–H]^−^	C_36_H_30_O_16_ (−0.4)	Salvianolic acid B
3	7.84	267,332	9.24	<0.05	447.1286 [M+H]^+^	C_22_H_22_O_10_ (−1.1)	Tilianin
4	8.62	267,332	5.22	<0.05	533.1293 [M+H]^+^	C_25_H_24_O_13_ (−0.4)	Acacetin 7-*O*-(6-*O*-malonyl)-β-*D*-glucoside
5	9.06	267,332	0.37	<0.05	489.1394 [M+H]^+^	C_24_H_24_O_11_ (−0.6)	Acacetin 7-*O*-(2″-*O*-acetyl)-β-*D*-glucoside
6	9.78	267,332	0.45	<0.05	575.1399 [M+H]^+^	C_27_H_26_O_14_ (−0.3)	Acacetin 7-*O*-(2″-*O*-acetyl-6″-*O*-malonyl)-β-D-glucoside
7	10.85	267,332	1.97	10.2	285.0755 [M+H]^+^	C_16_H_12_O_5_ (−2.8)	Acacetin

^a^ Content expressed as mg of each compound equivalents per g of dry weight.

## Data Availability

Data is contained within the article.

## Supplementary Materials

The following supporting information can be downloaded at: https://www.mdpi.com/article/10.3390/foods12030573/s1, Figures S1−S4. ^1^H-NMR spectra of **1**–**7**. Figures S5−S8. ^13^C-NMR spectra of **1**–**7**. Figures S9−S12. Identification of **1**–**7** by UPLC-qTOf MS. Table S1. ^1^H and ^13^C-NMR assign of compounds (**1**–**7**).
