# Supplementary material for: Potent Xanthine Oxidase Inhibitory Activity of Constituents of Agastache rugosa (Fisch. and C.A.Mey.) Kuntze
Source: Foods. 2023 Jan 28;12(3):573. doi: 10.3390/foods12030573 (PMC9914411; doi:10.3390/foods12030573)
Supplement: Supplementary file 1 [file foods-12-00573-s001.zip › foods-2177256-supplementary.pdf]

Supplementary Materials

Potent xanthine oxidase inhibitory activity of constituents of *Agastache rugosa* (Fisch. & C.A.Mey.) Kuntze

Heung Joo Yuk [1], HyungWon Ryu [2],* and Dong-Seon Kim [1],*

Title page ……………………………………………………………………………………… 1

Figure S1−4. ^{1}H-NMR spectrum of compounds (**1**−**7**) ……………………………………………… 2−8

Figure S5−8. ^{13}C-NMR spectrum of compounds (**1**−**7**) ……………………………………………… 9−15

Figure S9−12. Identification of compounds (**1**−**7**) by UPLC-Q-TOF MS ………………………………… 16−22

Table S1. ^{1}H- and ^{13}C-NMR assign of compounds (**1**−**7**)……………………………………… 23

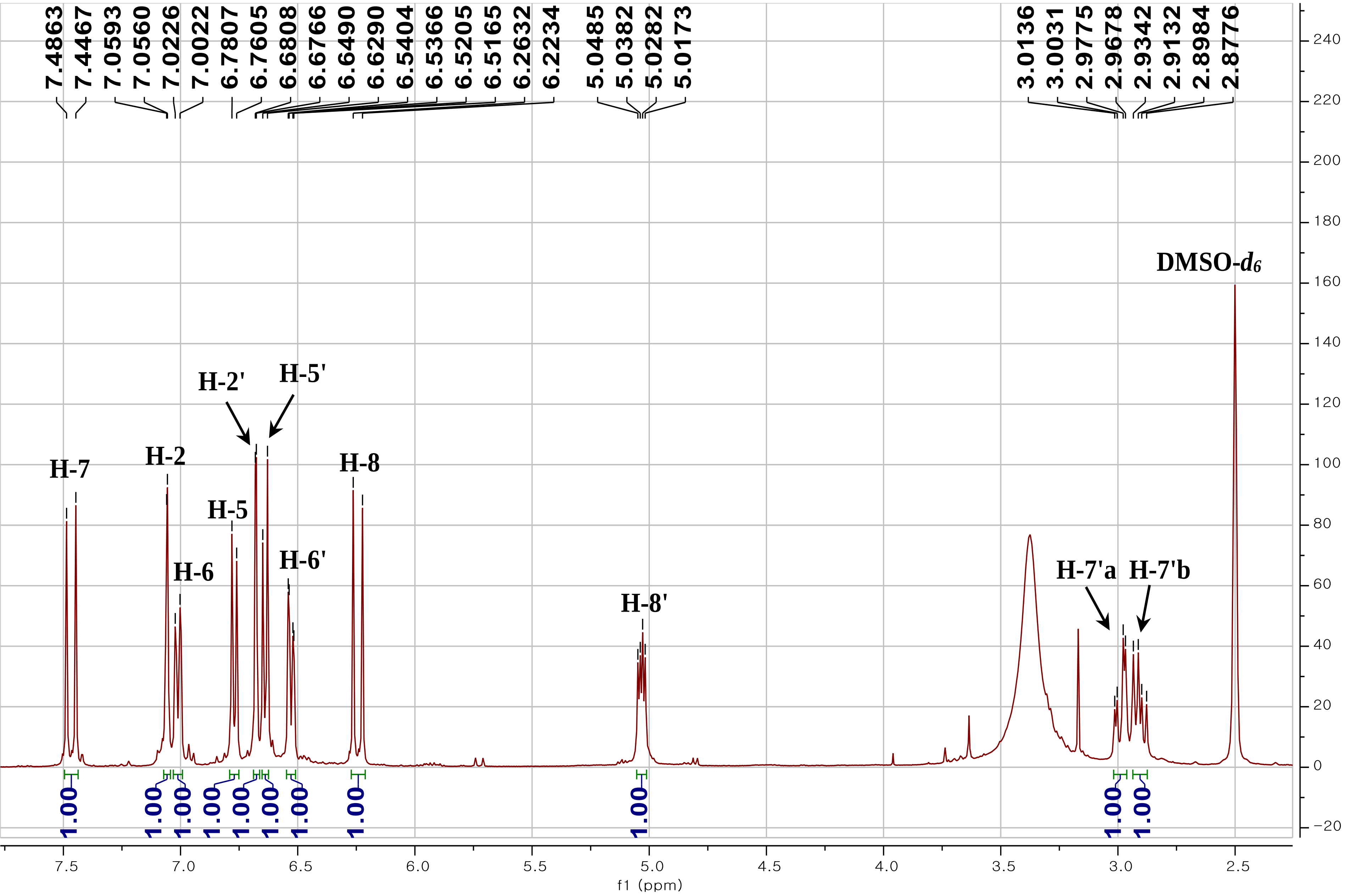

Figure S1. The ^{1}H NMR spectrum of compound **1** (400 MHz, DMSO-d_6).

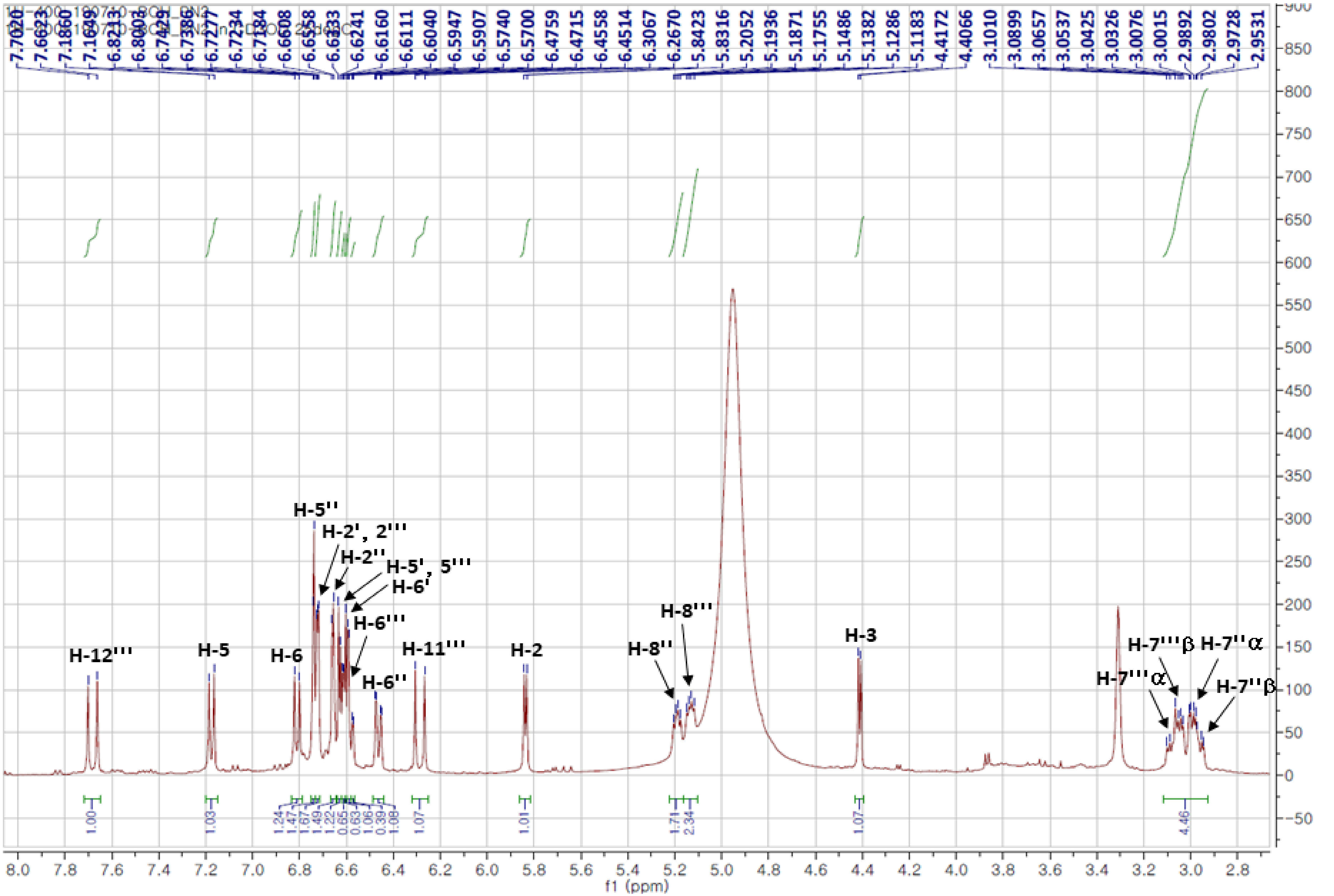

Figure S2. The ^{1}H NMR spectrum of compound **2** (500 MHz, CD_3OD).

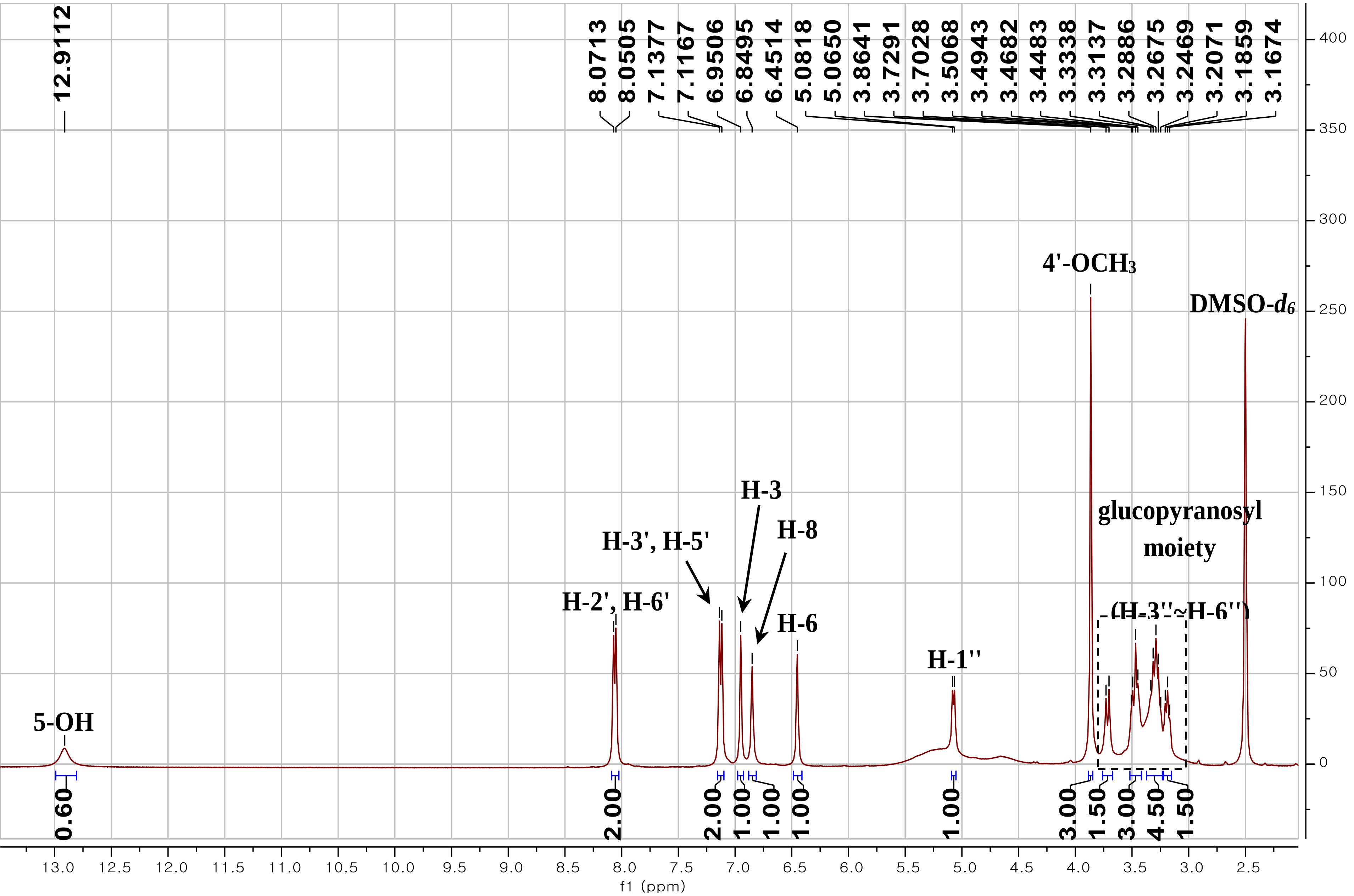

Figure S3. The ^{1}H NMR spectrum of compound **3** (400 MHz, DMSO-d_6).

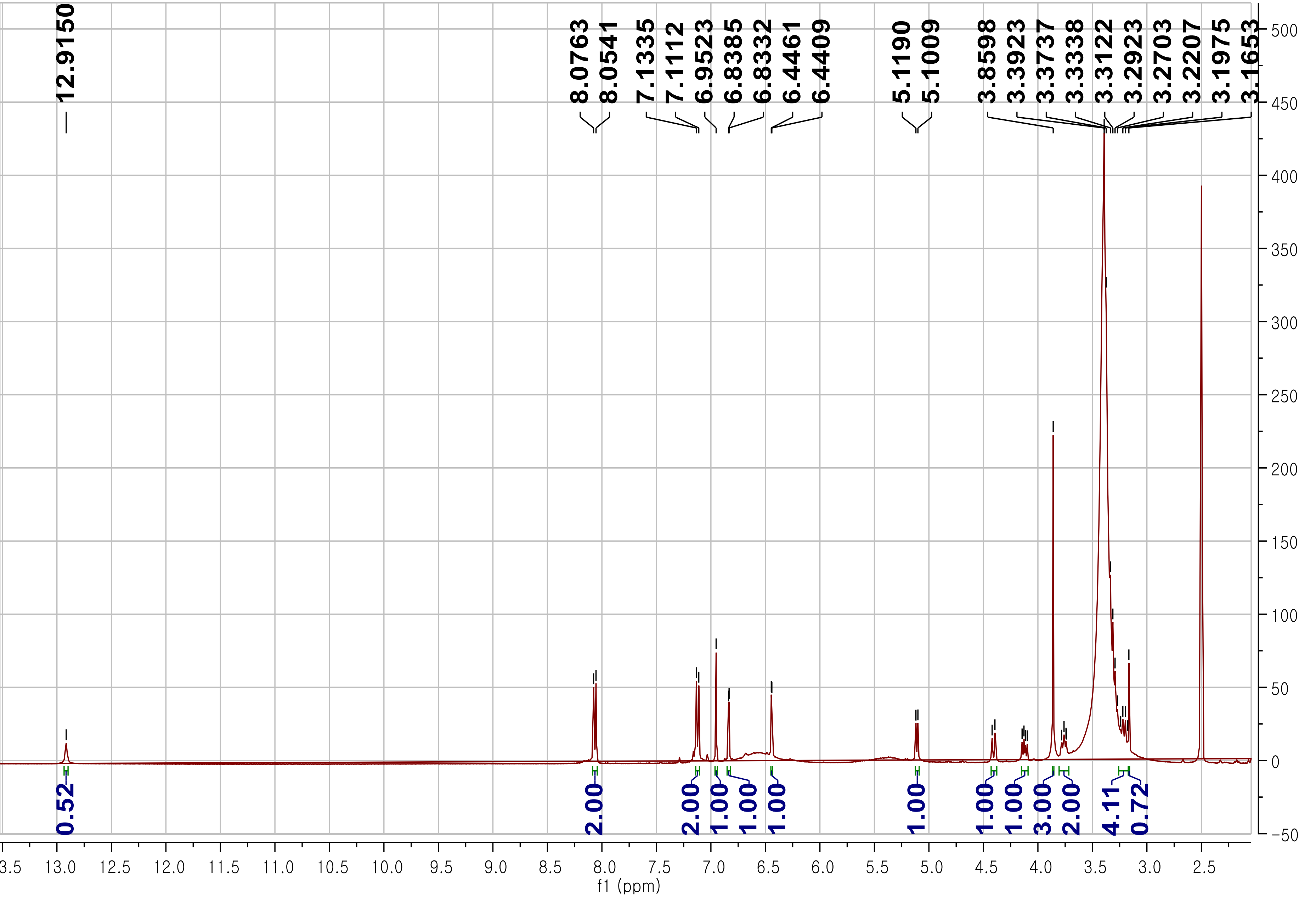

Figure S4. The ^{1}H NMR spectrum of compound **4** (400 MHz, DMSO-d_6).

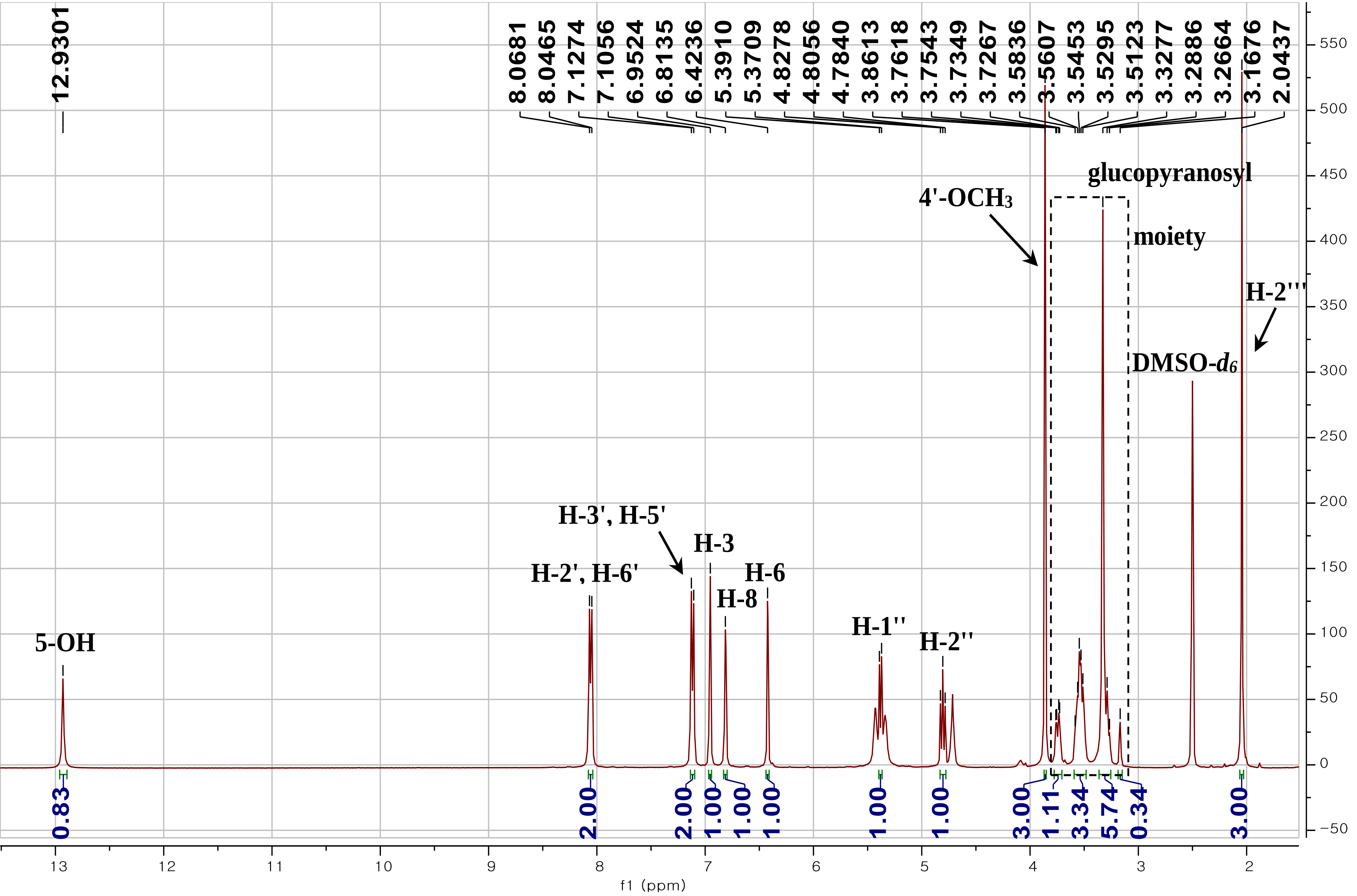

Figure S5. The ^{1}H NMR spectrum of compound **5** (400 MHz, DMSO-d_6).

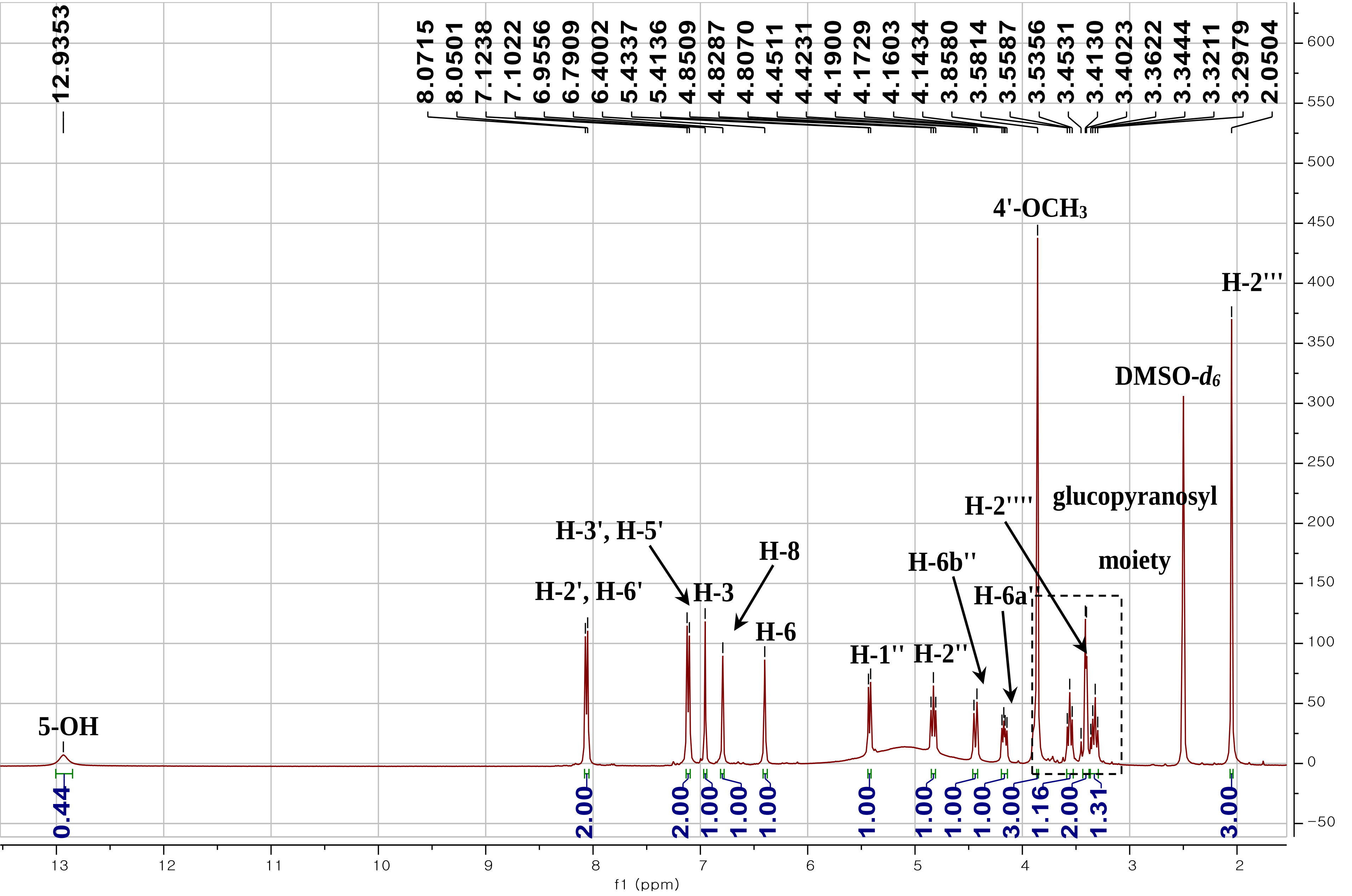

Figure S6. The ^{1}H NMR spectrum of compound **6** (400 MHz, DMSO-d_6).

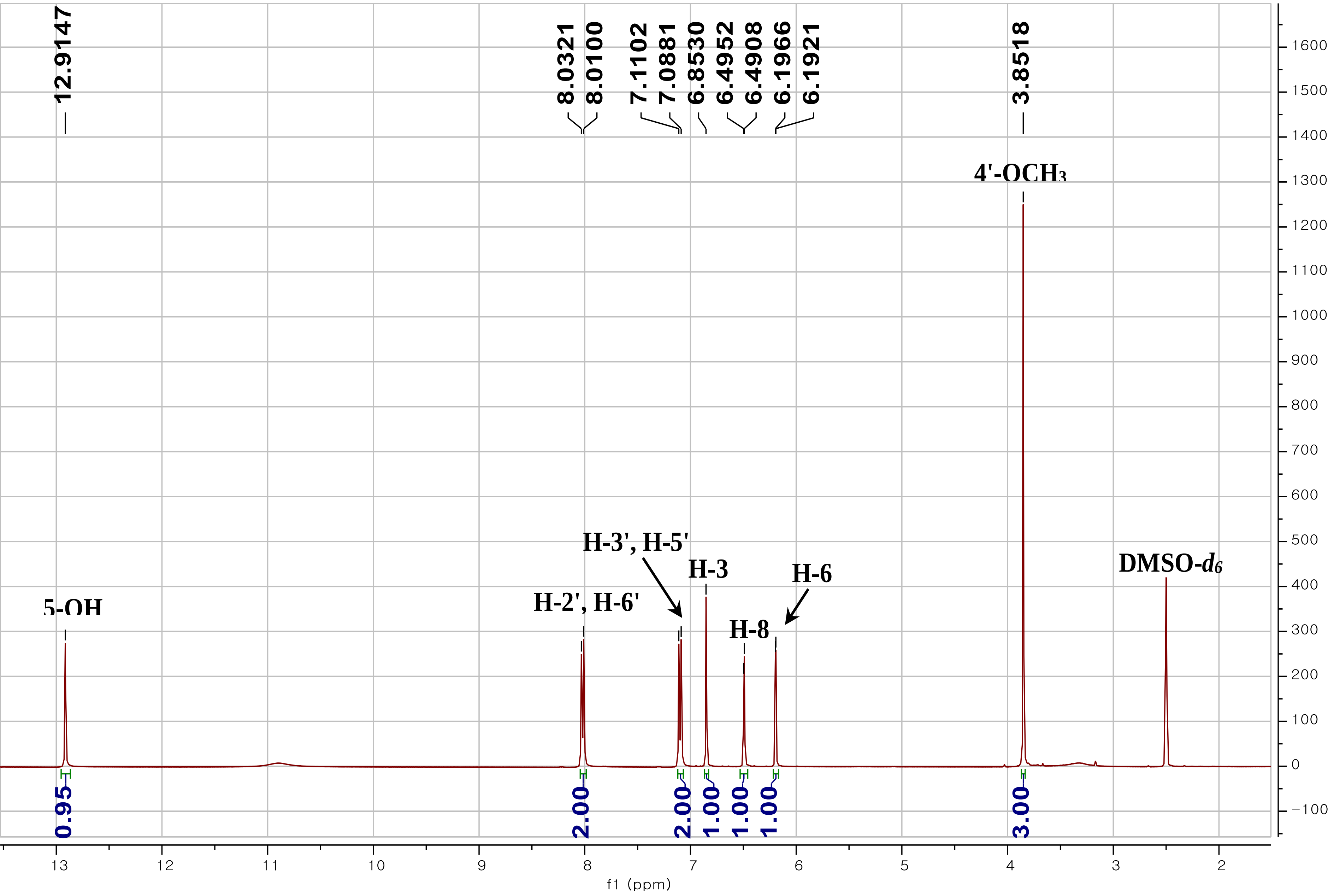

Figure S7. The ^{1}H NMR spectrum of compound **7** (400 MHz, DMSO-d_6).

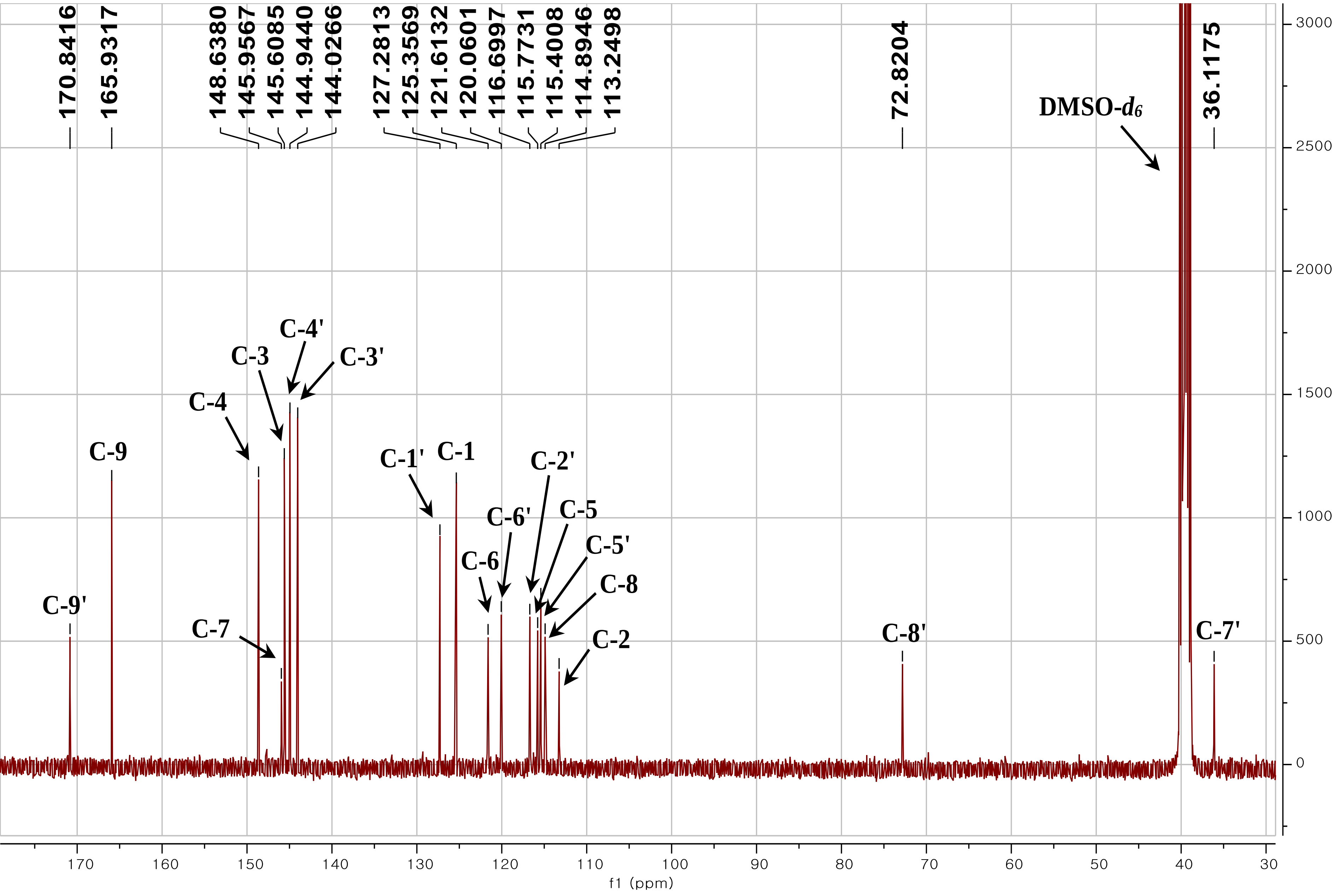

Figure S8. The ^{13}C NMR spectrum of compound **1** (100 MHz, DMSO-d_6).

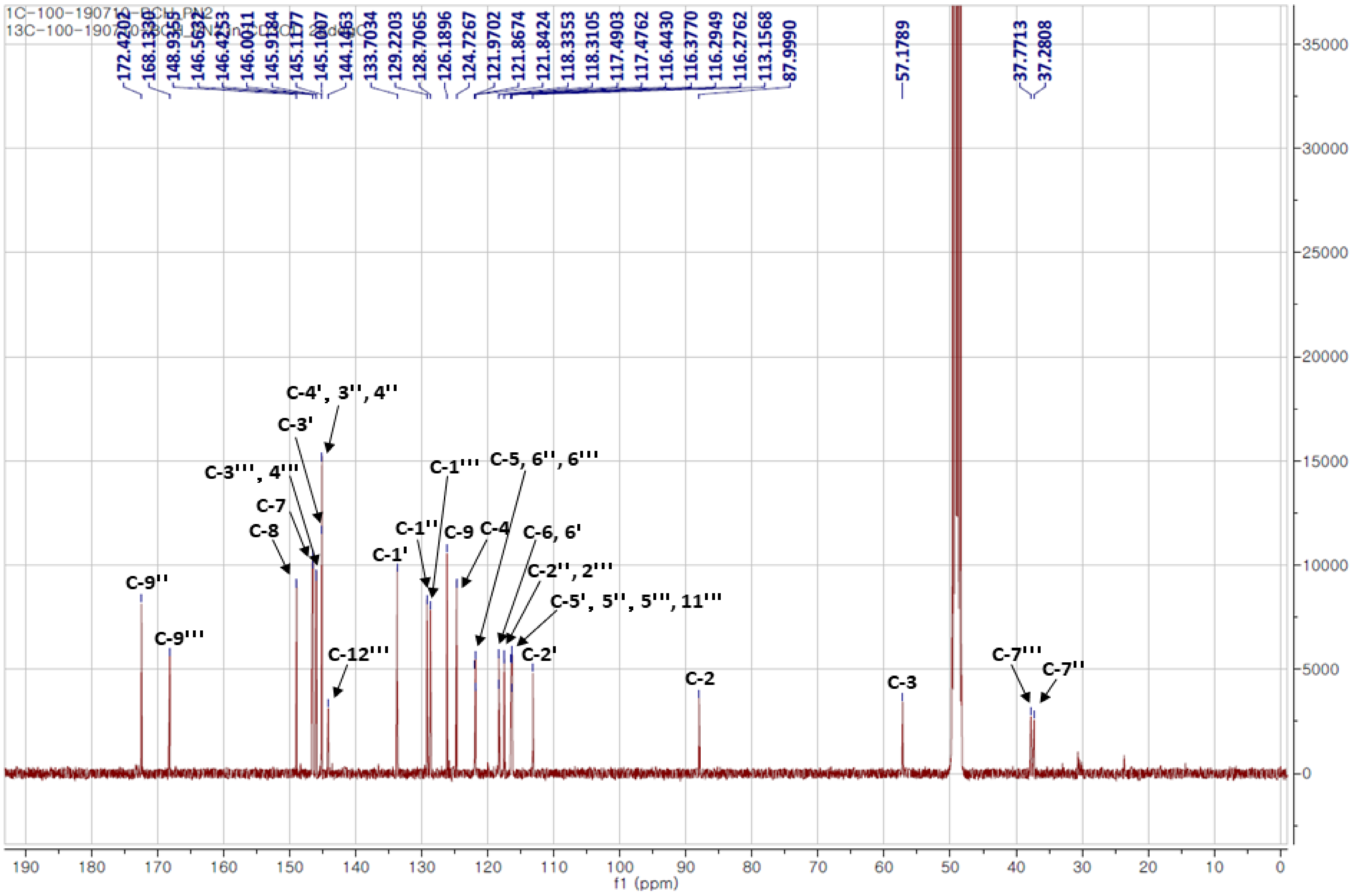

Figure S9. The ^{13}C NMR spectrum of compound **2** (125 MHz, CD_3OD).

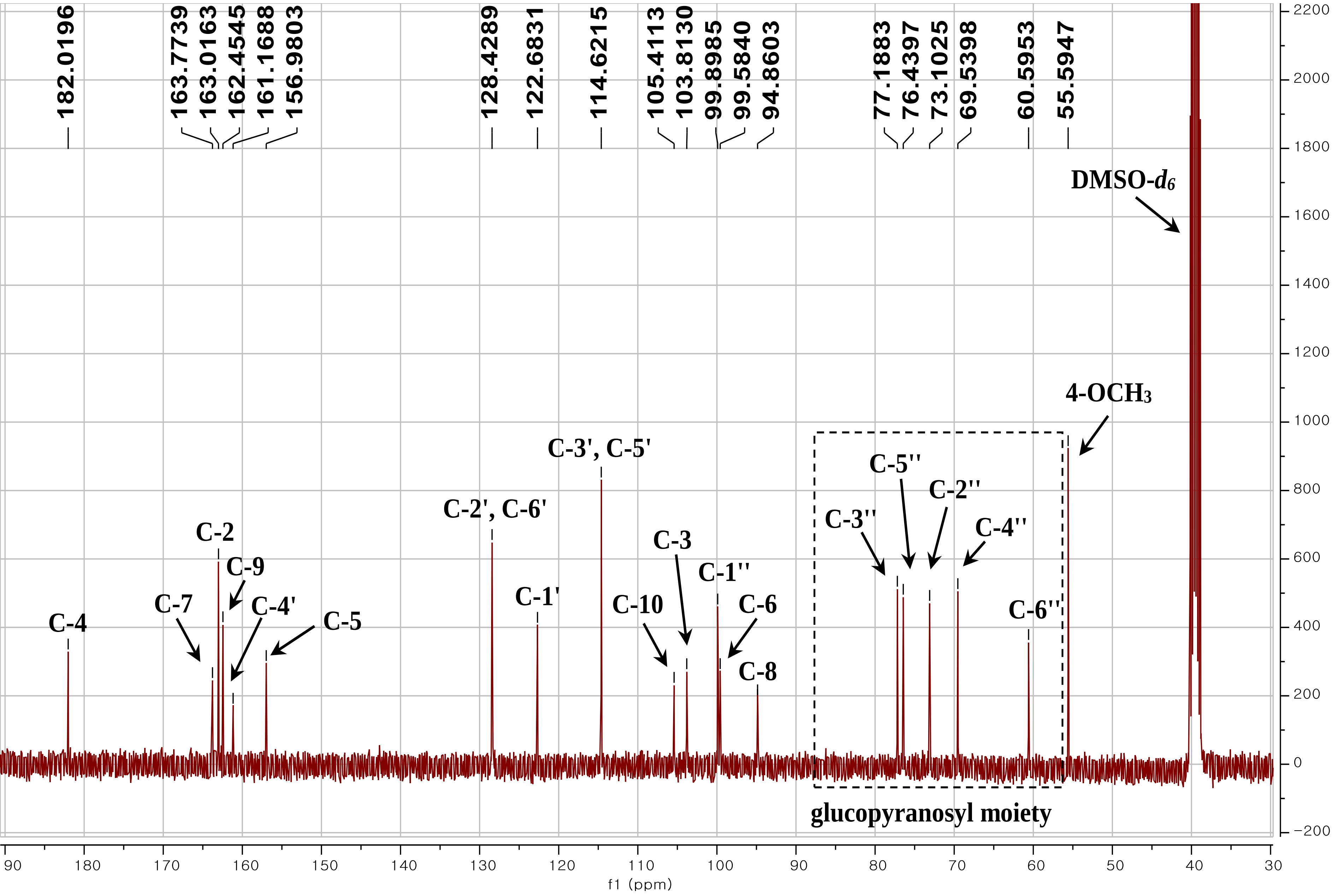

Figure S10. The ^{13}C NMR spectrum of compound **3** (100 MHz, DMSO-d_6).

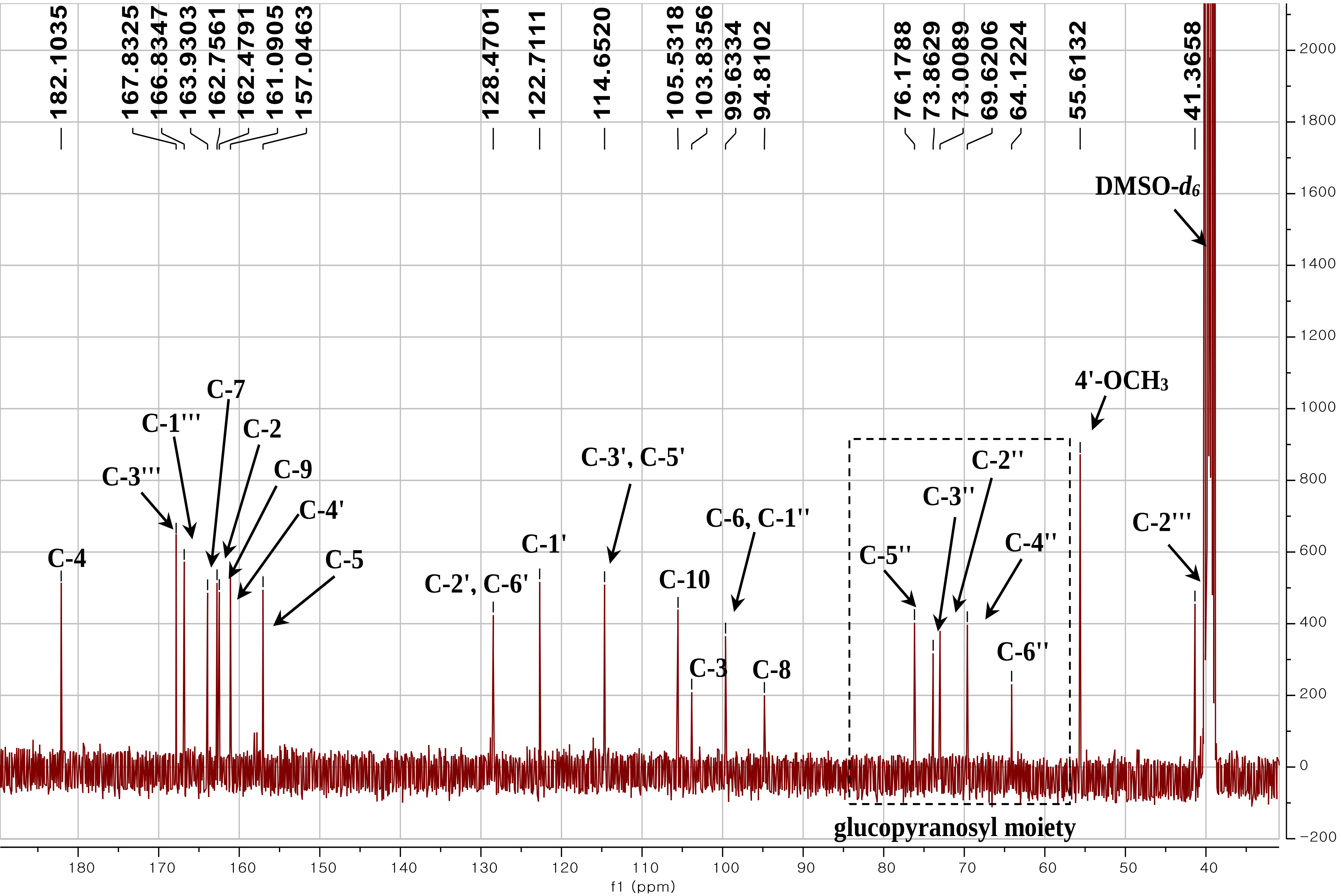

Figure S11. The ^{13}C NMR spectrum of compound **4** (100 MHz, DMSO-d_6).

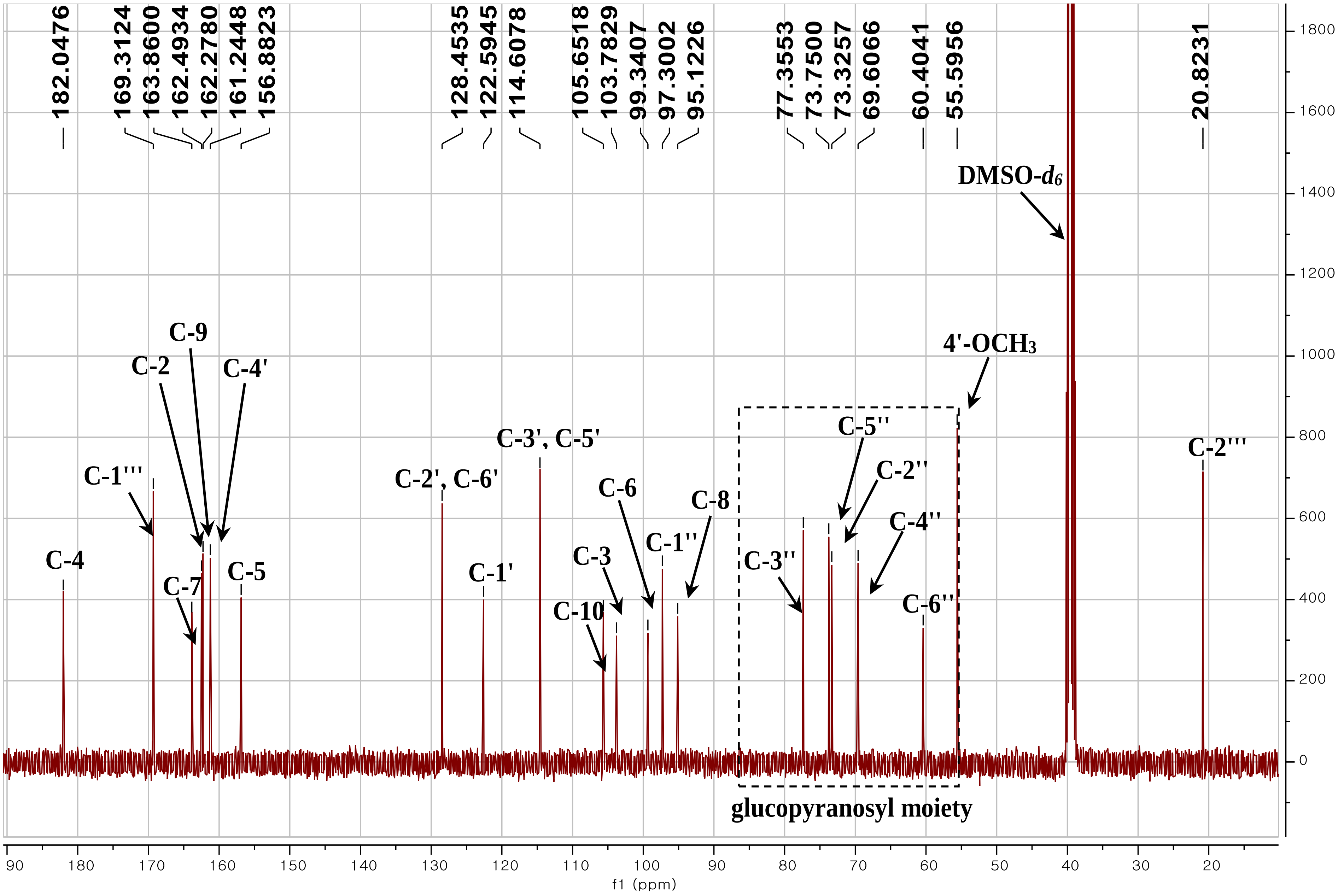

Figure S12. The ^{13}C NMR spectrum of compound **5** (100 MHz, DMSO-d_6).

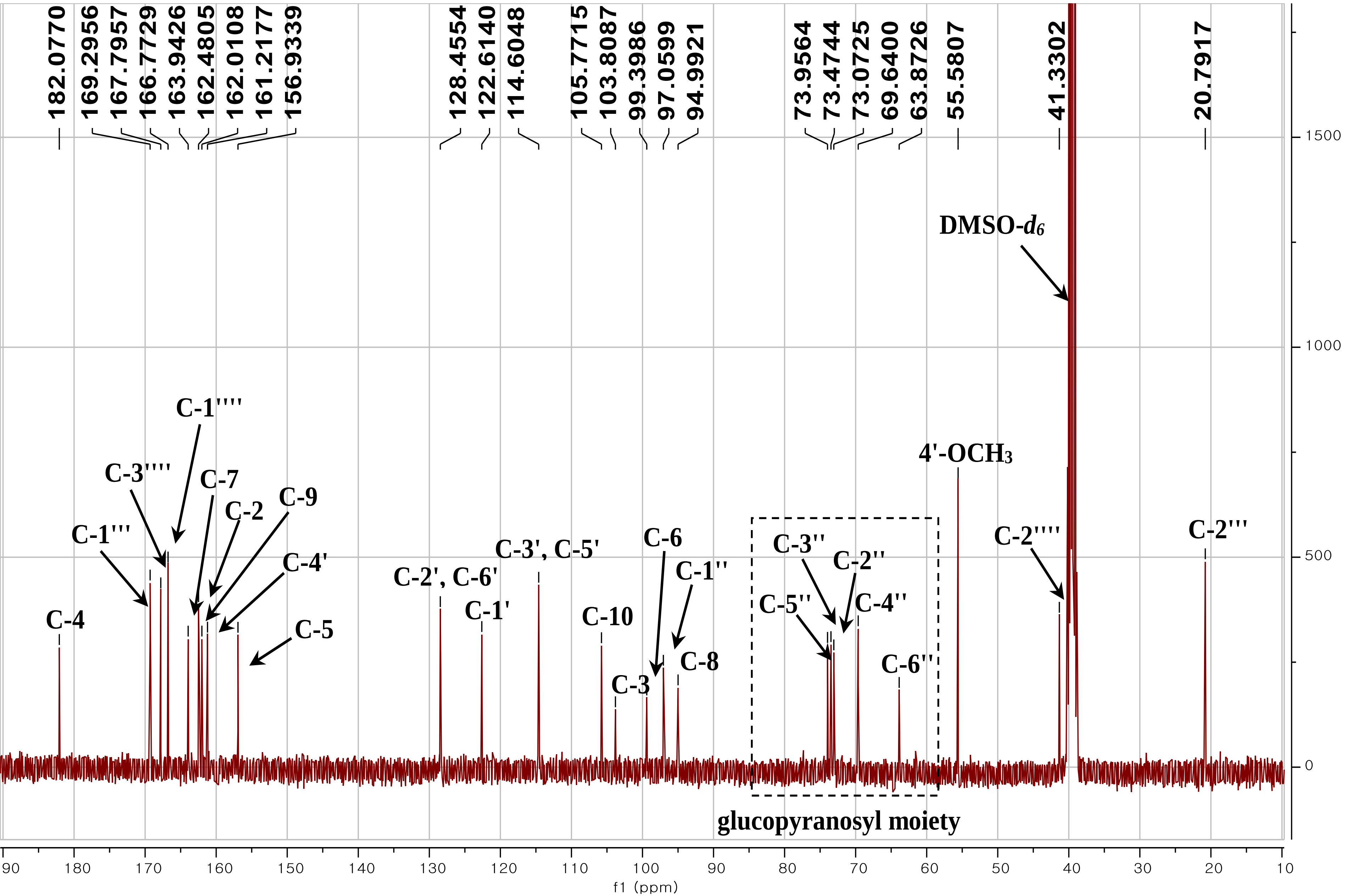

Figure S13. The ^{13}C NMR spectrum of compound **6** (100 MHz, DMSO-d_6).

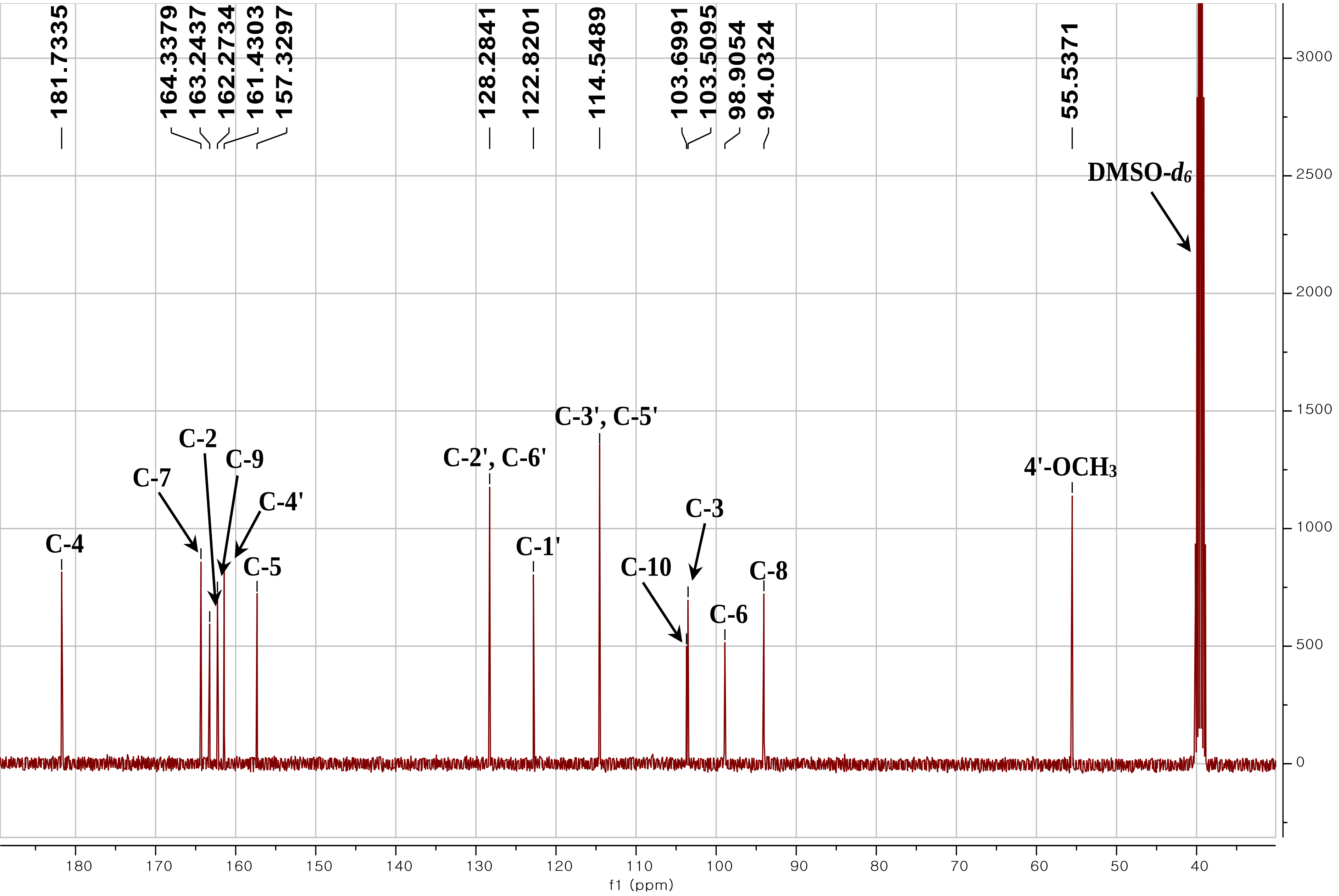

Figure S14. The ^{13}C NMR spectrum of compound **7** (100 MHz, DMSO-d_6).

[UV spectrum]

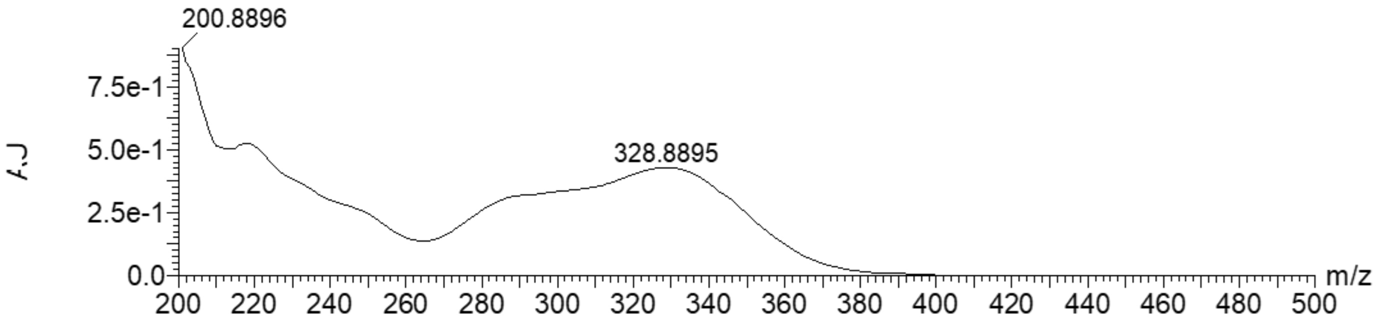

[MS Chromatogram]
negative

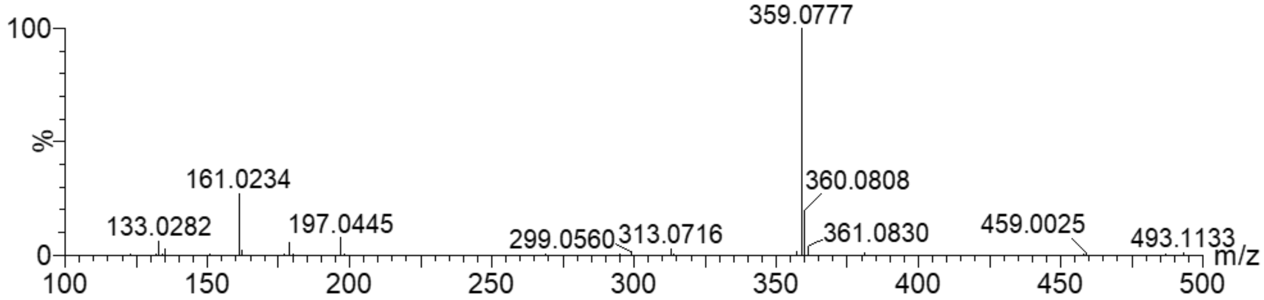

[MS/MS Chromatogram]
negative

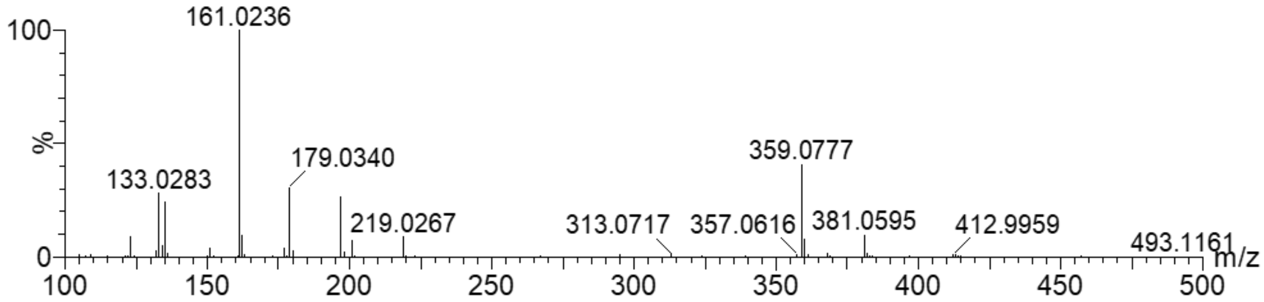

Elemental Composition:
[HR-ESI/MS] negative

Single Mass Analysis
Tolerance = 5.0 PPM / DBE: min = -1.5, max = 50.0
Element prediction: Off
Number of isotope peaks used for i-FIT = 3
Monoisotopic Mass, Even Electron Ions
76 formula(e) evaluated with 1 results within limits (up to 50 closest results for each mass)
Elements Used:

Mass	Calc. Mass	mDa	PPM	DBE	Formula	i-FIT	i-FIT Norm	Fit Conf %	C	H	O
359.0777	359.0767	1.0	2.8	11.5	C18 H15 O8	844.2	n/a	n/a	18	15	8

Figure S15. Identification of compound **1** by UPLC-QTof MS.

[UV spectrum]

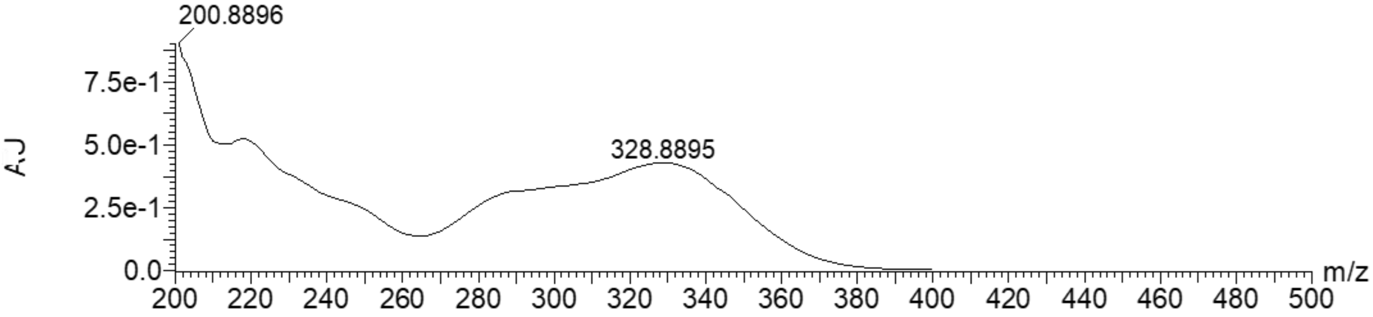

[MS Chromatogram]
negative

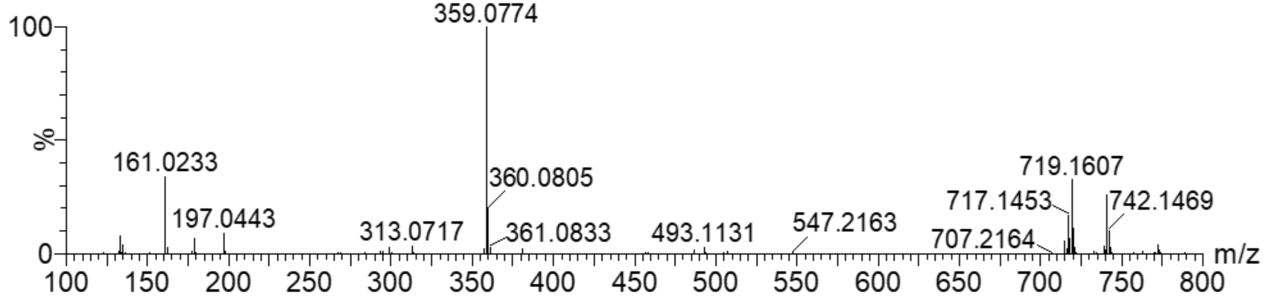

[MS/MS Chromatogram]
negative

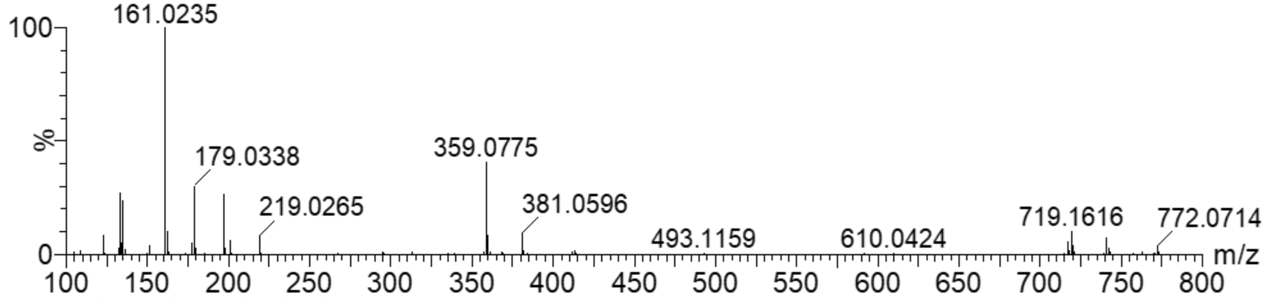

Elemental Composition:
[HR-ESI/MS] negative

Single Mass Analysis
Tolerance = 5.0 PPM / DBE: min = -1.5, max = 50.0
Element prediction: Off
Number of isotope peaks used for i-FIT = 3
Monoisotopic Mass, Even Electron Ions
237 formula(e) evaluated with 2 results within limits (up to 50 closest results for each mass)
Elements Used:

Mass	Calc. Mass	mDa	PPM	DBE	Formula	i-FIT	i-FIT Norm	Fit Conf %	C	H	O
717.1453	717.1456	-0.3	-0.4	22.5	C36 H29 O16	324.0	0.350	70.47	36	29	16
	717.1421	3.2	4.5	0.5	C18 H37 O29	324.9	1.220	29.53	18	37	29

Figure S16. Identification of compound **2** by UPLC-QTof MS.

[UV spectrum]

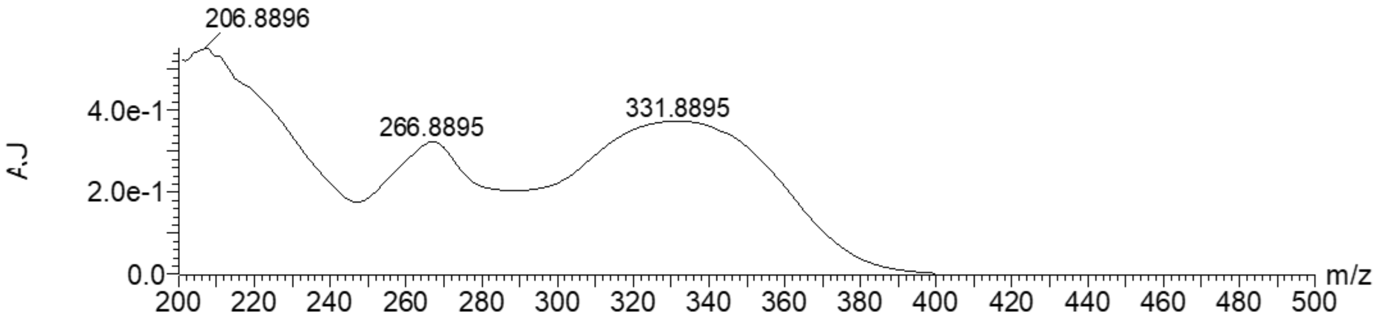

[MS Chromatogram]
positive

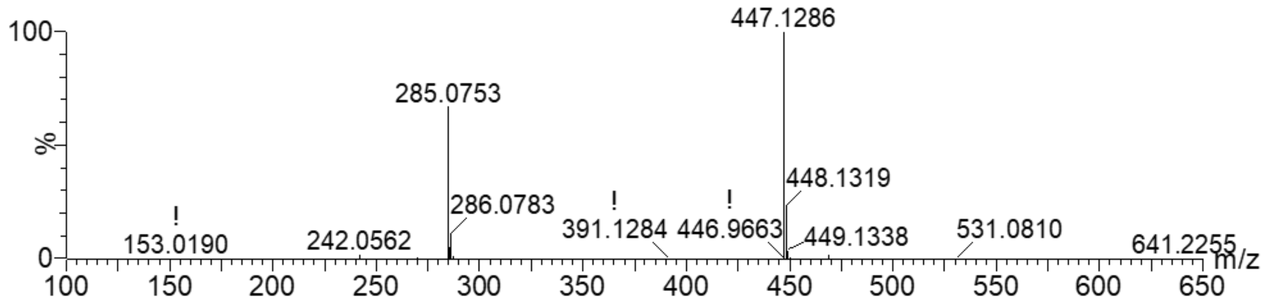

[MS/MS Chromatogram]
positive

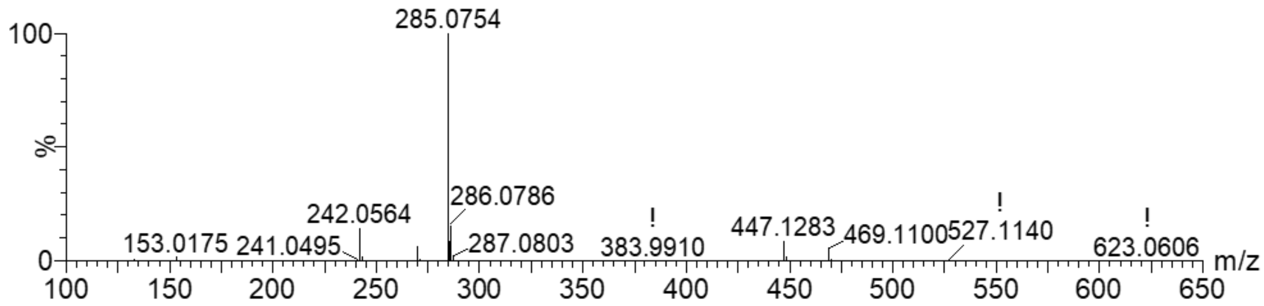

Elemental Composition:
[HR-ESI/MS] positive

Single Mass Analysis
Tolerance = 5.0 PPM / DBE: min = -1.5, max = 50.0
Element prediction: Off
Number of isotope peaks used for i-FIT = 3
Monoisotopic Mass, Even Electron Ions
108 formula(e) evaluated with 1 results within limits (up to 50 closest results for each mass)
Elements Used:

Mass	Calc. Mass	mDa	PPM	DBE	Formula	i-FIT	i-FIT Norm	Fit Conf %	C	H	O
447.1286	447.1291	-0.5	-1.1	11.5	$C_{22}H_{23}O_{10}$	554.5	n/a	n/a	22	23	10

Figure S17. Identification of compound **3** by UPLC-QTof MS.

[UV spectrum]

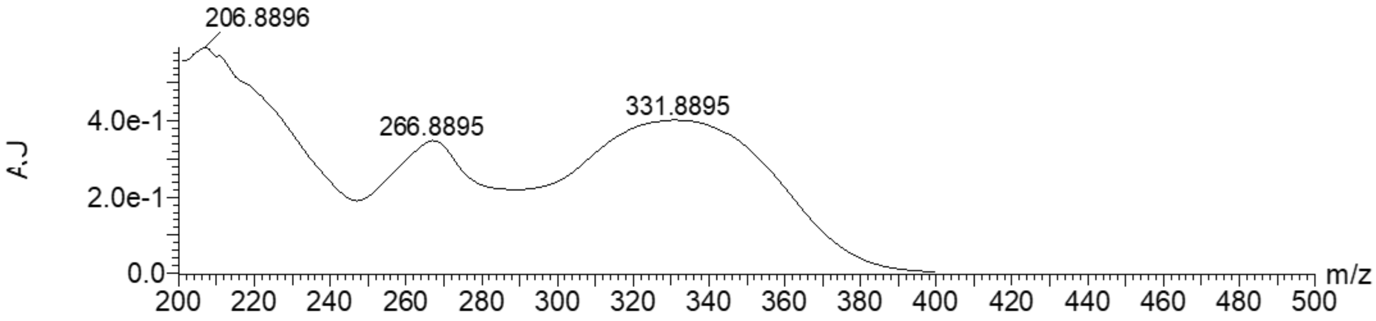

[MS Chromatogram]
positive

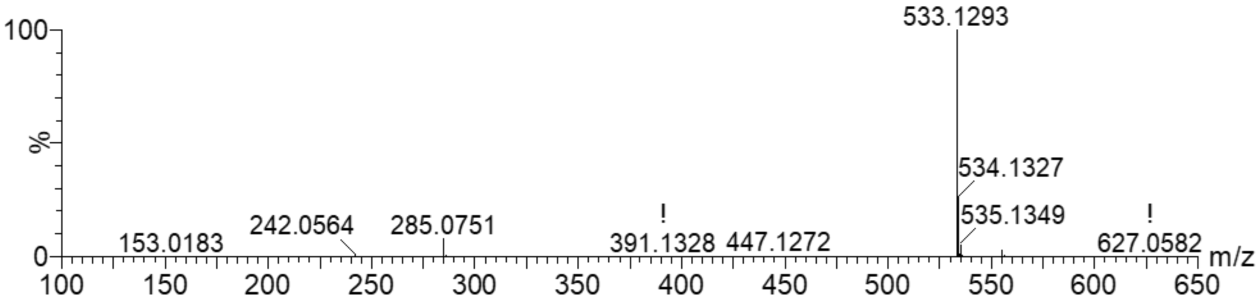

[MS/MS Chromatogram]
positive

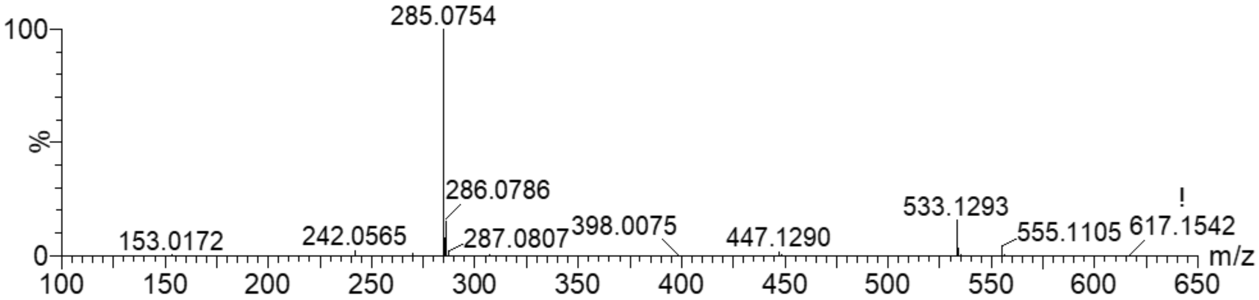

Elemental Composition:
[HR-ESI/MS] positive

Single Mass Analysis
Tolerance = 5.0 PPM / DBE: min = -1.5, max = 50.0
Element prediction: Off
Number of isotope peaks used for i-FIT = 3
Monoisotopic Mass, Even Electron Ions
141 formula(e) evaluated with 1 results within limits (up to 50 closest results for each mass)
Elements Used:

Mass	Calc. Mass	mDa	PPM	DBE	Formula	i-FIT	i-FIT Norm	Fit Conf %	C	H	O
533.1293	533.1295	-0.2	-0.4	13.5	C25 H25 O13	669.7	n/a	n/a	25	25	13

Figure S18. Identification of compound **4** by UPLC-QTof MS.

[UV spectrum]

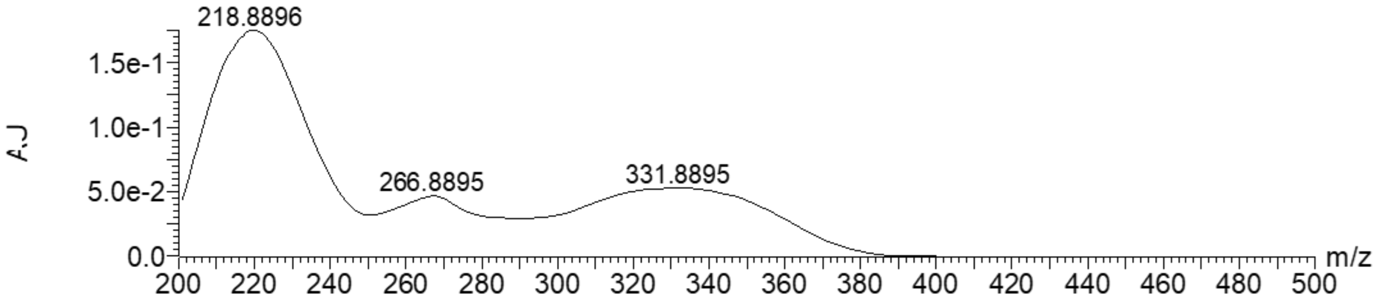

[MS Chromatogram]
positive

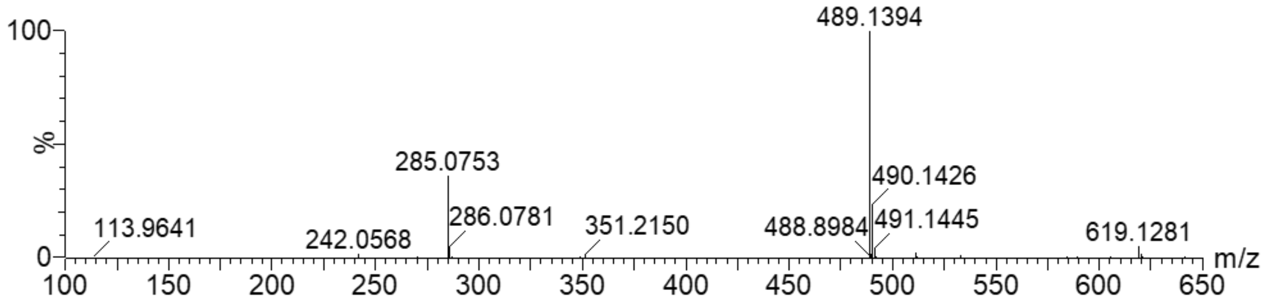

[MS/MS Chromatogram]
positive

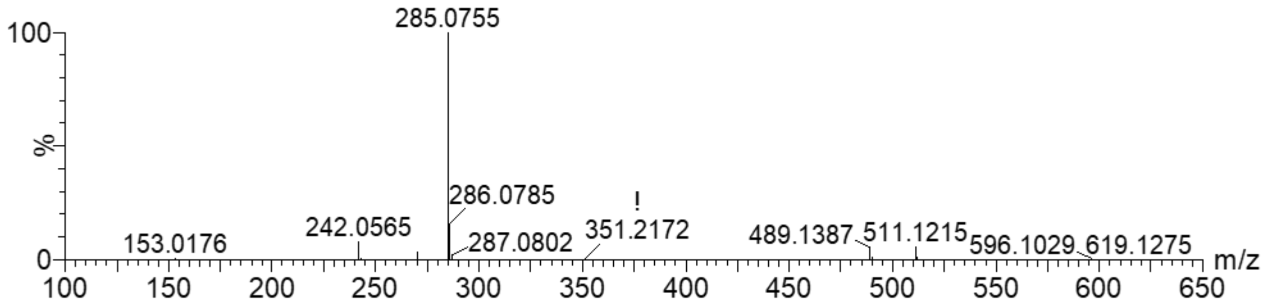

Elemental Composition:
[HR-ESI/MS] positive

Single Mass Analysis
Tolerance = 5.0 PPM / DBE: min = -1.5, max = 50.0
Element prediction: Off
Number of isotope peaks used for i-FIT = 3
Monoisotopic Mass, Even Electron Ions
121 formula(e) evaluated with 1 results within limits (up to 50 closest results for each mass)
Elements Used:

Mass	Calc. Mass	mDa	PPM	DBE	Formula	i-FIT	i-FIT Norm	Fit Conf %	C	H	O
489.1394	489.1397	-0.3	-0.6	12.5	C24 H25 O11	326.7	n/a	n/a	24	25	11

Figure S19. Identification of compound **5** by UPLC-QTof MS.

[UV spectrum]

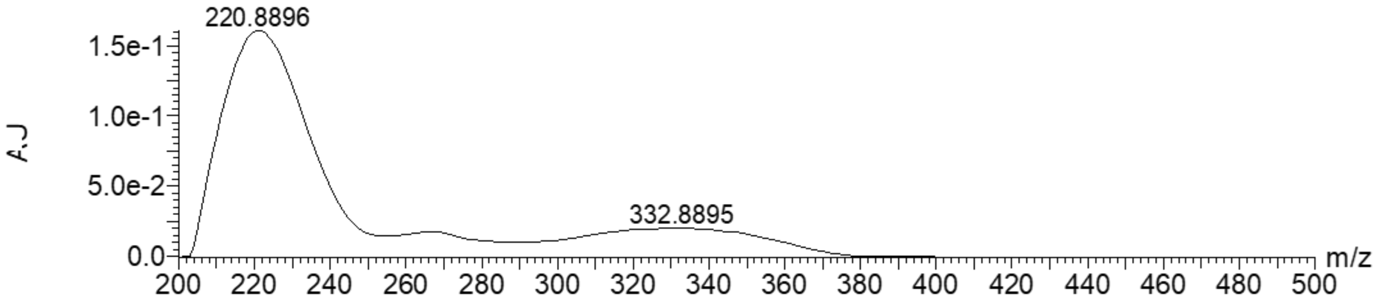

[MS Chromatogram]
positive

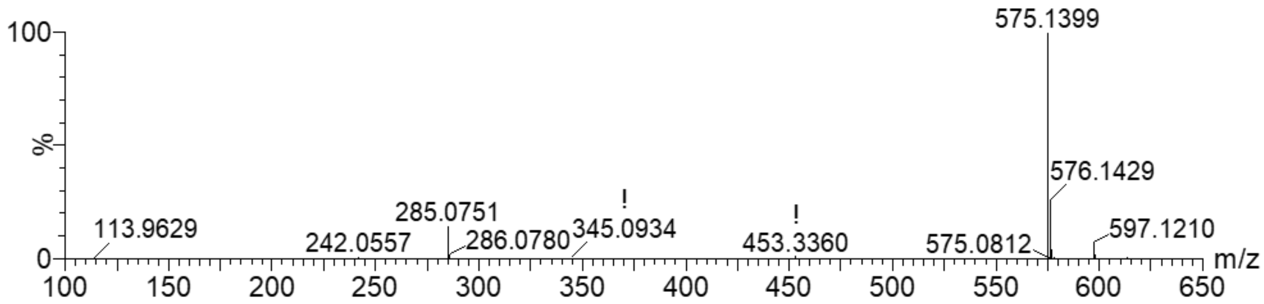

[MS/MS Chromatogram]
positive

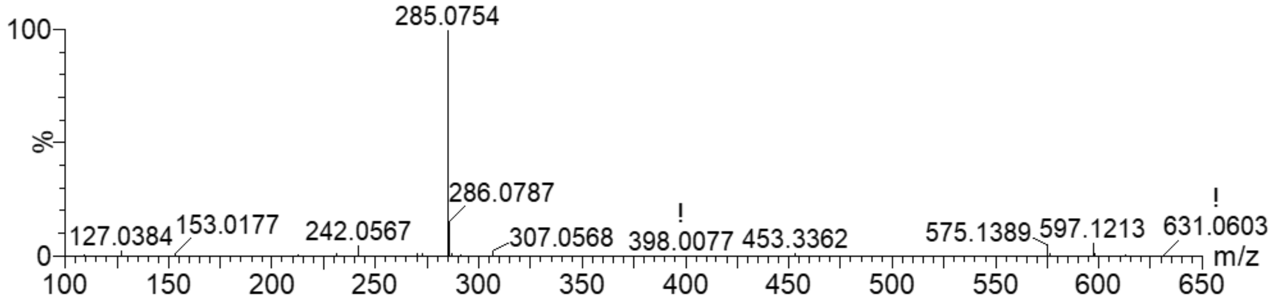

Elemental Composition:
[HR-ESI/MS] positive

Single Mass Analysis
Tolerance = 5.0 PPM / DBE: min = -1.5, max = 50.0
Element prediction: Off
Number of isotope peaks used for i-FIT = 3
Monoisotopic Mass, Even Electron Ions
166 formula(e) evaluated with 1 results within limits (up to 50 closest results for each mass)
Elements Used:

Mass	Calc. Mass	mDa	PPM	DBE	Formula	i-FIT	i-FIT Norm	Fit Conf %	C	H	O
575.1399	575.1401	-0.2	-0.3	14.5	C27 H27 O14	353.2	n/a	n/a	27	27	14

Figure S20. Identification of compound **6** by UPLC-QTof MS.

[UV spectrum]

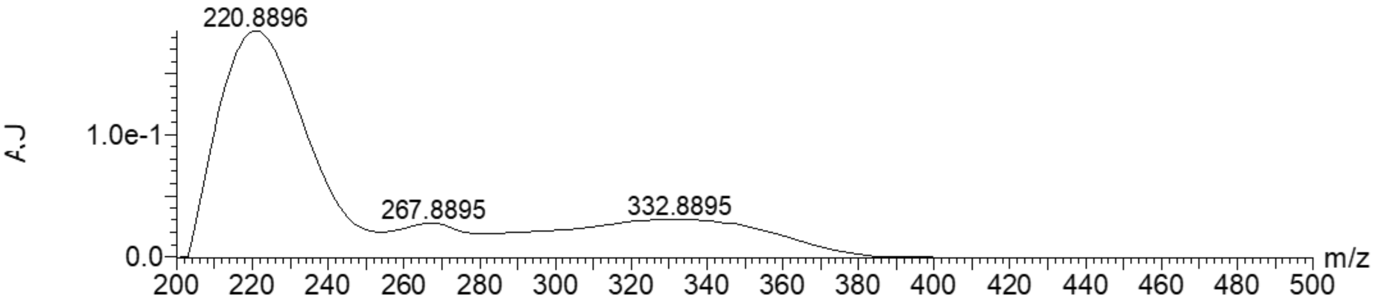

[MS Chromatogram]
positive

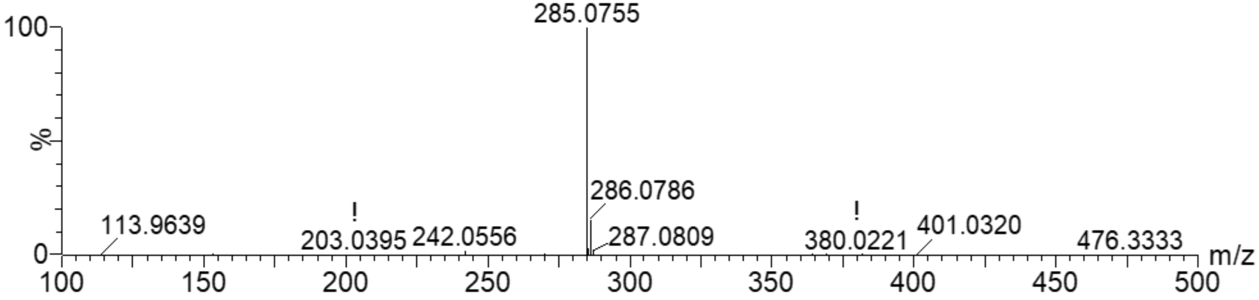

[MS/MS Chromatogram]
positive

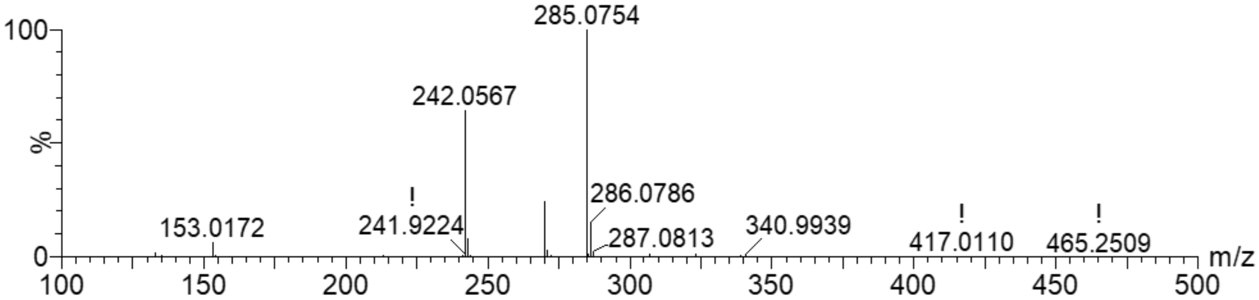

Elemental Composition:
[HR-ESI/MS] positive

Single Mass Analysis
Tolerance = 5.0 PPM / DBE: min = -1.5, max = 50.0
Element prediction: Off
Number of isotope peaks used for i-FIT = 3
Monoisotopic Mass, Even Electron Ions
48 formula(e) evaluated with 1 results within limits (up to 50 closest results for each mass)
Elements Used:

Mass	Calc. Mass	mDa	PPM	DBE	Formula	i-FIT	i-FIT Norm	Fit Conf %	C	H	O
285.0755	285.0763	-0.8	-2.8	10.5	C16 H13 O5	473.3	n/a	n/a	16	13	5

Figure S21. Identification of compound **7** by UPLC-QTof MS.

Table S1. ^{1}H- and ^{13}C-NMR assign of compounds (**1**–**7**).

Comp.	assign
**1**	^{1}H NMR (400 MHz, DMSO-d_6) δ_H 2.88 (1H, dd, J = 8.2 Hz, 8.2 Hz, H-7'b), 2.99 (1H, dd, J = 14.2 Hz, 4.2 Hz, H-7'a), 5.03 (1H, dd, J = 8.2 Hz, 4.2 Hz, H-8'), 6.24 (1H, d, J = 16.0 Hz, H-8), 6.53 (1H, dd, J = 8.0 Hz, 1.6 Hz, H-6'), 6.64 (1H, d, J = 8.0 Hz, H-5'), 6.68 (1H, d, J = 1.6 Hz, H-2'), 6.77 (1H, d, J = 8.0 Hz, H-5), 7.01 (1H, dd, J = 8.0 Hz, 1.2 Hz, H-6), 7.06 (1H, d, J = 1.2 Hz, H-2), 7.47 (1H, d, J = 15.8 Hz, H-7). ^{13}C NMR (DMSO-d_6, 100 MHz) δ_C 36.12 (C-7'), 72.82 (C-8'), 113.25 (C-2), 114.89 (C-8), 115.40 (C-5'), 115.77 (C-5), 116.70 (C-2'), 120.06 (C-6'), 121.61 (C-6), 125.36 (C-1), 127.28 (C-1'), 144.03 (C-3'), 144.94 (C-4'), 145.61 (C-3), 145.96 (C-7), 148.64 (C-4), 165.93 (C-9), 170.84 (C-9').
**2**	^{1}H NMR (500 MHz, CD_3OD) δ_H 3.10-2.95 (4H, m, H-7''α, 7''β, 7'''α, 7'''β), 4.40 (1H, d, J = 4.4 Hz, H-3), 5.11-5.20 (2H, m, H-8'', 8'''), 5.83 (1H, d, J = 4.4 Hz, H-2), 6.29 (1H, d, J = 16.0 Hz, H-11'''), 6.46 (1H, dd, J = 2.0, 8.0 Hz, H-6''), 6.57-6.65 (8H, m, H-6''', 6', 5', 5''', 2'', 2', 2''', 5''), 6.76 (1H, d, J = 8.4 Hz, H-6), 7.17 (1H, d, J = 16.0 Hz, H-5), 7.68 (1H, d, J = 16.0 Hz, H-12'''). ^{13}C NMR (125 MHz, CD_3OD) δ_C 172.4 (C-9''), 170.1 (C-10''), 168.1 (C-9'''), 166.9 (C-10'''), 148.9 (C-8), 146.5 (C-7), 146.4 (C-3''', 4'''), 145.9 (C-3', 4'), 145.1 (C-3'',4''), 133.7 (C-1'), 129.2 (C-1''), 128.7 (C-1'''), 126.2 (C-9), 124.7 (C-4), 144.1 (C-12'''), 121.9 (C-5, 6'', 6'''), 118.3 (C-6, 6'), 117.5 (C-2'', 2'''), 116.3 (C-5', 5'', 5''', 11'''), 113.2 (C-2'), 88.8 (C-2), 74.4 (C-8''), 74.4 (C-8''), 57.2 (C-3), 37.8 (C-7'''), 37.2 (C-7'').
**3**	^{1}H NMR (400 MHz, DMSO-d_6) δ_H 3.16-3.72 (6H, m, Glc-H), 3.86 (3H, s, 4'-OCH_3), 5.07 (1H, d, J = 7.6 Hz, H-1''), 6.45 (1H, d, J = 2.0 Hz, H-6), 6.85 (1H, d, J = 2.0 Hz, H-8), 6.95(1H, s, H-3), 7.13 (2H, d, J = 8.0 Hz, H-3', H-5'), 8.06 (2H, d, J = 8.0 Hz, H-2', H-6'), 12.91 (1H, br s, 5-OH). ^{13}C NMR (DMSO-d_6, 100 MHz) δ_C 55.59 (4'-OCH_3), 60.60 (C-6''), 69.54 (C-4''), 73.10 (C-2''), 76.44 (C-5''), 77.19 (C-3''), 94.86 (C-8), 99.58 (C-6), 99.90 (C-1''), 103.81(C-3), 105.41 (C-10), 114.62 (C-3', C-5'), 122.68 (C-1'), 128.43 (C-2', C-6'), 156.98 (C-5), 161.17 (C-4'), 162.45 (C-9), 163.02 (C-2), 163.77 (C-7), 182.02 (C-4).
**4**	^{1}H NMR (400 MHz, DMSO-d_6) δ_H 3.15-3.80 (4H, m, Glc-H), 3.39 (2H, s, H-2'''), 3.85 (3H, s, 4'-OCH_3), 4.12 (1H, dd, J = 11.8 Hz, 6.8 Hz, H-6''a), 4.41 (1H, dd, J = 11.8 Hz, 1.6 Hz, H-6''b), 5.11 (1H, d, J = 7.2 Hz, H-1''), 6.44 (1H, d, J = 2.0 Hz, H-6), 6.84 (1H, d, J = 2.0 Hz, H-8), 6.95 (1H, s, H-3), 7.12 (2H, d, J = 8.8 Hz, H-3', H-5'), 8.07 (2H, d, J = 8.8 Hz, H-2', H-6'), 12.92 (1H, br s, 5-OH). ^{13}C NMR (DMSO-d_6, 100 MHz) δ_C 41.37 (C-2'''), 55.61 (4'-OCH_3), 64.11 (C-6''), 69.61 (C-4''), 73.00 (C-2''), 73.85 (C-3''), 76.17 (C-5''), 94.80 (C-8), 99.62 (C-6, C-1''), 103.40 (C-3), 105.53 (C-10), 112.70 (C-1'), 114.64 (C-3', C-5'), 128.46 (C-2', C-6'), 157.04 (C-5), 161.08 (C-4'), 162.47 (C-9), 162.75 (C-2), 163.92 (C-7), 166.82 (C-1'''), 167.82 (C-3'''), 182.09 (C-4).
**5**	^{1}H NMR (400 MHz, DMSO-d_6) δ_H 2.04 (3H, s, H-2'''), 3.15-3.80 (5H, m, Glc-H), 3.86 (3H, s, 4'-OCH_3), 4.81 (1H, dd, J = 9.4 Hz, 8.2 Hz, H-2''), 5.39 (1H, d, J = 7.6 Hz, H-1''), 6.42 (1H, d, J = 2.0 Hz, H-6), 6.81 (1H, d, J = 2.0 Hz, H-8), 6.95 (1H, s, H-3), 7.12 (2H, d, J = 8.8 Hz, H-3', H-5'), 8.06 (2H, d, J = 8.8 Hz, H-2', H-6'), 12.93 (1H, br s, 5-OH). ^{13}C NMR (DMSO-d_6, 100 MHz) δ_C 20.82 (C-2'''), 55.60 (4'-OCH_3), 60.40 (C-6''), 69.60 (C-4''), 73.33 (C-2''), 73.75 (C-5''), 77.36 (C-3''), 95.12 (C-8), 97.30 (C-1''), 99.34 (C-6), 103.78 (C-3), 105.65 (C-10), 114.61 (C-3', C-5'), 122.59 (C-1'), 128.45 (C-2', C-6'), 156.88 (C-5), 161.24 (C-4'), 162.28 (C-9), 162.49 (C-2), 163.86 (C-7), 169.31 (C-1'''), 182.04 (C-4).
**6**	^{1}H NMR (400 MHz, DMSO-d_6) δ_H 2.05 (3H, s, H-5'''), 3.30-3.95 (3H, m, Glc-H), 3.41 (2H, s, H-2''''), 3.86 (3H, s, 4'-OCH_3), 4.17 (1H, dd, J = 11.0 Hz, 6.8 Hz, H-6''a), 4.44 (1H, d, J = 11.0 Hz, H-6''b), 4.83 (1H, m, H-2''), 5.42 (1H, d, J = 8.0 Hz, H-1''), 6.40 (1H, d, J = 2.0 Hz, H-6), 6.79 (1H, d, J = 2.0 Hz, H-8), 6.96 (1H, s, H-3), 7.12 (2H, d, J = 8.8 Hz, H-3', H-5'), 8.06 (2H, d, J = 8.8 Hz, H-2', H-6'), 12.94 (1H, br s, 5-OH). ^{13}C NMR (DMSO-d_6, 100 MHz) δ_C 20.79 (C-2'''), 41.33 (C-2''''), 55.58 (4'-OCH_3), 63.87 (C-6''), 69.64 (C-4''), 73.07 (C-2''), 73.47 (C-3''), 73.95 (C-5''), 94.99 (C-8), 97.06 (C-1''), 99.40 (C-6), 103.81 (C-3), 105.77 (C-10), 114.60 (C-3', C-5'), 122.61 (C-1'), 128.46 (C-2', C-6'), 156.93 (C-5), 161.22 (C-4'), 162.01 (C-9), 162.48 (C-2), 163.94 (C-7), 166.77 (C-1''''), 167.79 (C-3''''), 169.30 (C-1'''), 182.09 (C-4).
**7**	^{1}H NMR (400 MHz, DMSO-d_6,) δ_H 3.85 (3H, s, 4'-OCH_3), 6.19 (1H, d, J = 1.8 Hz, H-6), 6.49 (1H, d, J = 1.8 Hz, H-8), 6.85 (1H, s, H-3), 7.10 (2H, d, J = 8.8 Hz, H-3', H-5'), 8.02 (2H, d, J = 8.8 Hz, H-2', H-6'), 12.92 (1H, br s, 5-OH). ^{13}C NMR (DMSO-d_6, 100 MHz) δ_C 55.54 (4'-OCH_3), 94.03 (C-8), 98.91 (C-6), 103.51 (C-3), 103.70 (C-10), 114.55 (C-3', C-5'), 122.82 (C-1'), 128.28 (C-2', C-6'), 157.33 (C-5), 161.43 (C-4'), 162.27 (C-9), 163.24 (C-2), 164.34 (C-7), 181.73 (C-4).
